# An Alternative Splicing Program for Mouse Craniofacial Development

**DOI:** 10.3389/fphys.2020.01099

**Published:** 2020-09-04

**Authors:** Joan E. Hooper, Kenneth L. Jones, Francis J. Smith, Trevor Williams, Hong Li

**Affiliations:** ^1^Department of Cell and Developmental Biology, University of Colorado School of Medicine, Aurora, CO, United States; ^2^Department of Pediatrics, Section of Hematology, Oncology, and Bone Marrow Transplant, University of Colorado School of Medicine, Aurora, CO, United States; ^3^Department of Craniofacial Biology, University of Colorado School of Dental Medicine, Aurora, CO, United States

**Keywords:** facial development, ectoderm, mesenchyme, nasal epithelium, facial prominences, skipped exon, RNA binding proteins, splicing regulators

## Abstract

Alternative splicing acts as a fundamental mechanism to increase the number of functional transcripts that can be derived from the genome – and its appropriate regulation is required to direct normal development, differentiation, and physiology, in many species. Recent studies have highlighted that mutation of splicing factors, resulting in the disruption of alternative splicing, can have profound consequences for mammalian craniofacial development. However, there has been no systematic analysis of the dynamics of differential splicing during the critical period of face formation with respect to age, tissue layer, or prominence. Here we used deep RNA sequencing to profile transcripts expressed in the developing mouse face for both ectodermal and mesenchymal tissues from the three facial prominences at critical ages for facial development, embryonic days 10.5, 11.5, and 12.5. We also derived separate expression data from the nasal pit relating to the differentiation of the olfactory epithelium for a total of 60 independent datasets. Analysis of these datasets reveals the differential expression of multiple genes, but we find a similar number of genes are regulated only via differential splicing, indicating that alternative splicing is a major source of transcript diversity during facial development. Notably, splicing changes between tissue layers and over time are more prevalent than between prominences, with exon skipping the most common event. We next examined how the variation in splicing correlated with the expression of RNA binding proteins across the various datasets. Further, we assessed how binding sites for splicing regulatory molecules mapped with respect to intron exon boundaries. Overall these studies help define an alternative splicing regulatory program that has important consequences for facial development.

## Introduction

Human craniofacial development is a complex process and frequently goes awry to cause a major class of birth defects, orofacial clefting (Dixon et al., 2011). Proper facial development requires three sets of prominences coming together by growth, morphogenesis, and fusion (Supplementary Figure 1): paired maxillary prominences (MxP) and mandibular prominences (MdP), and a frontonasal prominence (FNP), which includes both lateral and medial nasal processes separated by the nasal pits (Jiang et al., 2006). Facial prominences are composed of an outer layer of ectoderm and a large core of neural crest- and mesoderm-derived mesenchymal cells. Signaling crosstalk between these two tissue layers is critical for facial morphogenesis (Chai and Maxson, 2006). One of our goals stemming from the FaceBase Consortium^1^ (Brinkley et al., 2016; Samuels et al., in press) was to develop a comprehensive description of gene expression and gene regulation during a critical phase of development, using the mouse as the best available model system to stand in for the human. Previously, we and others have examined gene expression using microarray analysis (Gong et al., 2005; Feng et al., 2009; Brunskill et al., 2014; Hooper et al., 2017) or single cell RNA-seq (Li et al., 2019) to obtain an overview of general and specific expression patterns. However, none of these techniques allowed a comprehensive analysis of alternative splicing and how the regulation of alternative splicing occurs in face formation.

Alternative splicing (AS) is a crucial process which allows for an expansion of the repertoire of transcripts made from the genome. This process is critical in gene expression regulation and increasing proteome diversity. It affects approximately 90–95% of genes in mammals and contributes to cell lineage determination and differentiation, acquisition and maintenance of cell identity, and multiple developmental processes (Pan et al., 2008; Wang et al., 2008; Baralle and Giudice, 2017). It has become clear that alternative splicing in different tissues is regulated by coordinated networks of splicing regulators (Ule and Blencowe, 2019). The importance of alternative splicing in craniofacial development is demonstrated by the observation that abnormalities in spliceosome or splicing regulators occur in several human birth defects often involving the craniofacial complex (Bain et al., 2016; Marques et al., 2016; Loiselle and Sutherland, 2018; Berube-Simard and Pilon, 2019; Beauchamp et al., 2020; Griffin and Saint-Jeannet, 2020). Two splicing regulators involved in promoting differential splicing in the ectoderm – Esrp1/2 – are known to cause mouse orofacial clefting of the lip and palate when deleted (Warzecha et al., 2009; Bebee et al., 2015; Lee et al., 2020). In addition, deletion of splicing factor *Rbfox2* in neural crest leads to craniofacial defects including cleft palate (Cibi et al., 2019). However, there is as yet no systematic analysis of differential splicing during face formation.

Therefore, we performed RNA-seq on both ectodermal and mesenchymal tissues derived from the three types of facial prominence at three critical stages, E10.5, E11.5, and E12.5, during which these structures align and fuse together to form the lower jaw and upper face. The sensitivity of this deep sequencing technique allowed us to expand our analysis of differential gene expression: to tissues and timepoints not possible with microarray approaches; to address the development and differentiation of the nasal pit olfactory epithelium as a separate entity from the FNP ectoderm and mesenchyme; and to identify potential differences in alternative splicing. In the context of alternative splicing, we describe the variations in alternative splicing that occur across time, tissue layer and prominence, and correlate these data with the differential expression of RNA binding proteins. Our findings greatly increase our understanding of the gene expression profiles and alternative splicing program occurring in these tissues, and provide insights into the regulation of alternative splicing that forms an essential part of transcript diversity during facial development.

## Materials and Methods

### Sample Preparation and RNA Sequencing

All mouse experiments were performed in accordance with protocols approved by the University of Colorado Anschutz Medical Campus Animal Care and Usage Committee. We used inbred C57BL/6J mice (stock #000664; The Jackson Laboratory) for all the RNA-seq experiments and outbred CD1 mice (Envigo) for *in situ* hybridization. Embryos were staged by a combination of embryonic day and morphological criteria. Facial prominences were dissected as described previously (Feng et al., 2009; Hooper et al., 2017) at E10.5, E11.5, and E12.5, according to the color-coded schematic shown in Supplementary Figure 1. Also note that by E12.5, the MxP and nasal processes are in the midst of fusing together, and therefore we used an established landmark, the nasolacrimal groove between row 3 and 4 of the vibrissae, to separate the derivatives of these two tissues (Wrenn and Wessells, 1984). The ectoderm of each facial prominence was then separated from the mesenchyme using a previously published protocol (Li and Williams, 2013). In addition, the nasal olfactory epithelium (NE) was separated from FNP surface ectoderm at E11.5 and E12.5. However, since the boundary between NE and surface ectoderm is not clear at E10.5, the NE and surface ectoderm of the FNP at E10.5 were collected as one sample. After dissection, all the tissues were submerged in RNAlater (Invitrogen, AM7021) at 4°C overnight then stored at −20°C. To obtain sufficient RNA, particularly for the ectodermal samples, we pooled tissues from an average of 38, 24, and 10 embryos per sample at E10.5, E11.5, and E12.5, respectively. Biological triplicates were collected for each tissue. Next, RNA was size-selected using the microRNA purification kit (Norgen, 21300) to extract fractions containing RNA larger than 200 nt or smaller than 200 nt according to the manufacturer’s instructions. The > 200 nt fractions were then used for RNA-seq analysis. RNA quality assessment, library preparation, and RNA-seq were carried out by the University of Colorado Genomics and Microarray Core Facility at the Anschutz Medical campus. Briefly, RNA integrity was measured using RNA ScreenTape (Agilent) and RNA integrity numbers of all the samples were found to be > 9.0. Given the minimal degradation revealed by the RNA integrity numbers, libraries were prepared with 1 ug of RNA from each sample for poly A selected RNA-seq by using TruSeq Stranded mRNA Sample Prep Kit (Illumina). All samples from each timepoint were processed simultaneously – 18 samples for E10.5 and 21 samples for both E11.5 and E12.5, due to the additional triplicate nasal epithelia samples – for a total of 60 samples. We performed paired-end 125 bp sequencing on the Illumina HiSEQ 2500 platform and obtained 130—250 million raw reads per sample.

### Data Analysis of the RNA-seq Data

#### Mapping and Differential Expression Analysis

For all the RNA-seq data, we first performed adapter and quality trimming (Phred score > 33) with Trimmomatic 0.36 on the raw FASTQ files before aligning to the mouse reference genome mm10 (Mus_musculus.GRCm38.79) with gSNAP (version 2014-12-17).^2^ Gene expression (FPKM) was quantified with Cufflinks v2.2.1.^3^ Sequence alignment/map files (.bam) of all the RNA-seq from this study are available via FaceBase (FB00000867). A gene-by-sample expression spreadsheet is shown in Supplementary Table 1. The fidelity of our tissue and prominence preparations were assessed using well-characterized gene expression markers (see Supplementary Figure 3 for examples). We used custom R scripts to analyze differential expression (Supplementary Table 2) across tissue layers, ages, and prominences by three-way analysis of variance (three-way ANOVA) as described previously (Hooper et al., 2017).

#### Analyzing Differentially Used Alternative Splicing With rMATS

Differentially used alternative splicing was analyzed with rMATS 3.2.5 (Shen et al., 2014)^4^ using the ENSEMBL gene models (Mus_musculus.GRCm38.86.gtf). rMATS detects alternative splicing events in categories of skipped exon (SE), mutually exclusive exon, alternative 5′ splice site, alternative 3′ splice site, and retained intron (shown as the schematics in Figure 2A). As the RNA-seq reads are relatively short (125 nt), these events do not translate to full length transcript isoforms, since an isoform can have multiple alternative splicing events, as well as different transcription start sites, and/or poly A addition sites. Instead, rMATS uses these short reads to compare local differences in specific regions of transcripts derived from a particular gene. Also note that the category mutually exclusive exon is not limited to mutually exclusive exon usage, but also records more complex splicing events as part of this category (Wang and Rio, 2018). Mapped .bam files were used as input for rMATS with default rMATS settings. For each alternative splicing event, this software calculates Percentage of splicing inclusion (PSI) for each sample across the biological triplicates, detects the differential PSI (ΔPSI) between two different conditions, and outputs two types of results, using reads mapped to splice junctions only or using reads mapped to both splice junctions and exon body. These two types of results are comparable. Therefore, we used results using reads mapped to both splice junctions and exon body in our study. Forty-five comparisons were performed (Supplementary Table 4) by holding two variables constant and varying the third variable across age, layer, and prominence. We used an FDR < 5% and | ΔPSI | ≥ 10% as significance cutoffs (Supplementary Data Sheet 1). To gain a systemic view of AS during facial development, we identified all the AS events in any comparison, retrieved the PSI values for these events in all comparisons, and then filtered the events with maximal PSI difference among all conditions > 0.1 (Supplementary Table 5) using custom python and R scripts.

#### Expression of RNA Binding Proteins (RBPs) During Facial Development

To assess the complexity of the AS regulatory program during facial development, we profiled the expression of the mouse homologs of a comprehensive list of human RBPs (Gerstberger et al., 2014) and a curated list of splicing regulators (Han et al., 2013; Bebee et al., 2015) with three-way ANOVA. The differentially expressed RBPs and splicing regulators are shown in Supplementary Tables 10, 11, respectively. Moreover, we cross-referenced the binding motifs of the differentially expressed splicing regulators between E11.5 MxP ectoderm and mesenchyme tissues (one-way ANOVA, adjust *p* < 0.01, fold change > 1.5) to the sequence within and around relevant skipped exons by feeding the rMATS SE output of the same comparison into rMAPS2 (Hwang et al., 2020) with default parameters.

#### Gene Annotation

Differentially expressed genes or the relevant genes of differentially used alternative splicing events were annotated by using Enrichr (Chen et al., 2013; Kuleshov et al., 2016).^5^ The significant enriched terms (adjusted *p* < 0.05) were shown in Supplementary Table 3 for differentially expressed genes and Supplementary Tables 6–8 for SE events.

### Validation of Alternative Splicing Discovered by RNA-seq

Primers used for making *in situ* probes are shown in Supplementary Table 9. *In situ* hybridization for E12.5 whole mount embryos and frozen sections were performed as described previously (Li et al., 2019).

BaseScope^TM^ analysis was performed to detect SE events with short exons. The BaseScope^TM^ probes for detecting both skipping and inclusion isoforms were custom made by Advanced Cell Diagnostics (Supplementary Table 9). BaseSope^TM^ analysis was performed on E11.5 frontal fixed frozen sections using BaseScope^TM^ Detection Reagent Kit v2-RED (Advanced Cell Diagnostics, 323910) according to the manufacturer’s recommendations with modified pretreatment for sections. Briefly, fixed frozen sections were washed with PBS for 5 minutes (min) at room temperature, baked at 60°C for 30 min, fixed in 4% PFA at room temperature for 90 min, dehydrated in 50, 70, 100, and 100% ethanol for 5 min at room temperature each, dried at 60°C for 10 min, treated with hydrogen peroxide (Advanced Cell Diagnostics, 322381) at room temperature for 10 min, and rinsed with distilled water once. Following acclimatization of the sections in boiling water for 10 s, target retrieval was performed in boiling 1X target retrieval buffer (Advanced Cell Diagnostics, 322000) in an Oster Steamer (Model 5715) for 5 min. After rinsing in distilled water briefly twice, the sections were washed in fresh 100% ethanol for 10 s and air dried. After creating and drying a barrier, the sections were treated with Protease III (Advanced Cell Diagnostics, 322381) at 40°C for 30 min, and then washed twice in distilled water followed by hybridization and washing according to the manufacturer’s instructions. Finally, slides were counterstained for 30 s with 50% hematoxylin (Sigma-Aldrich, GHS232-1L). BaseScope^TM^ signals are visible as red dots, while the tissues are counterstained blue.

For reverse transcription PCR experiments, tissues were collected and processed for RNA extraction as for RNA-seq analysis. cDNA was generated from 300 ng of RNA with the SuperScript^TM^ III First-Strand Synthesis Kit (Invitrogen, 18080-051). PCR was then conducted using the Taq DNA polymerase kit with Q-solution (Qiagen, 201205) with primers shown in Supplementary Table 9 spanning the skipped exons using an Eppendorf Mastercycler nexus Sx1. PCR products were separated on a 2% agarose gel.

## Results

### Highly Reproducible RNA-seq Dataset for Mouse Facial Development

**FIGURE 1:**
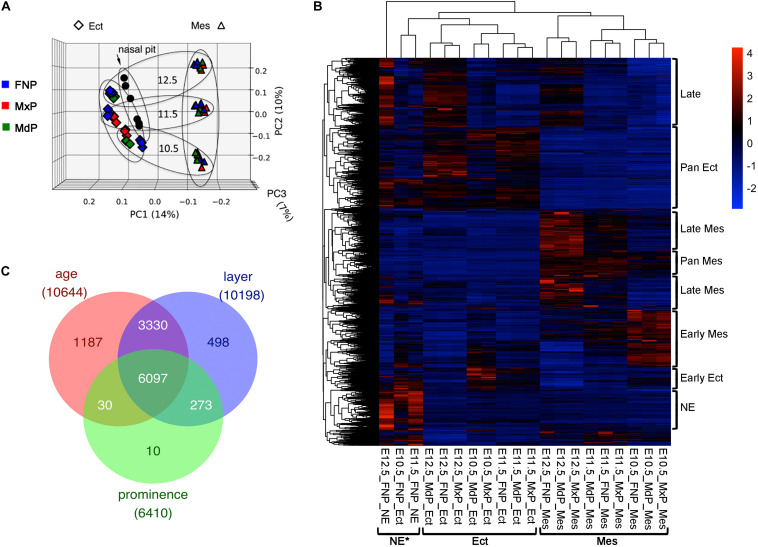
RNA-seq for the developing mouse face. **(A)** Principal component analysis of the RNA-seq data of 60 facial samples: ectoderm (rhombus), mesenchyme (triangle), nasal pit (circle), FNP (blue), MxP (red), and MdP (green). These samples separated first by tissue layers (PC1) and then by age (PC2). Note, PC3 does not separate by prominence. Larger ovals group the sample ages (more horizontal), or layers (more vertical). **(B)** Gene expression program for facial development. Heatmap of ∼11,000 differentially expressed genes (maximal average FPKM across all samples > 1, minimal adjusted *p*-value across all samples < 0.01, and maximal fold change across all samples > 2) after scaling with color key at upper right. Each row is a gene, and each column is a sample with mean expression from three biological replicates. The sample names are shown at the bottom. The samples are clustered mainly by tissue layers as bracketed at the bottom. The differential expressed gene programs are bracketed on the right side of the heatmap. GO terms enriched in these gene programs are shown in Supplementary Table 3. **(C)** Venn diagram showing differentially expressed genes by age, layer, and prominence. The number of shared or unique genes are labeled in the corresponding area. Ect, ectoderm; FNP, frontonasal process; MdP, mandibular prominence; Mes, mesenchyme; MxP, maxillary prominence; NE, nasal epithelium. *Note that the E10.5_FNP_Ect is a mixture of NE and FNP surface ectoderm.

To elucidate the transcriptomic and splicing programs for facial development, mRNA-seq analyses were performed for ectoderm and mesenchyme samples derived from the three sets of facial prominences (FNP, MxP, and MdP) at E10.5, E11.5, and E12.5. Because of the unique properties of the nasal/olfactory epithelium (NE), E11.5 and E12.5 FNP ectoderm samples were further subdivided into surface ectoderm and NE. Biological triplicates for each of the 20 conditions (60 samples) were sequenced at a depth compatible with isoform analysis (130–250 million 2 × 125 reads per sample). Principal component analysis shows high reproducibility among biological replicates, with the PC1 separating samples by tissue layer and PC2 separating them by age (Figure 1A). The gene expression data are presented in Supplementary Table 1. There are 17804 genes with average FPKM > 1 in at least one condition. Among these, 11444 genes are differentially expressed (DE) across tissue layers, ages, and/or prominences, as determined using three-way ANOVA with cutoffs set as minimal adjusted *p*-value < 0.01 and maximal fold change across all samples > 2 (Figures 1B,C and Supplementary Table 2). Consistent with the principal component analysis, the majority of the differential gene expression occurs between tissue layers or ages. This finding also agrees with our previous microarray-based analysis of the facial transcriptome (Hooper et al., 2017) and although these two datasets were derived by different approaches there is strong agreement concerning gene expression between the new RNA-seq data and these previous microarray studies (Supplementary Figures 2, 3A).

**FIGURE 2:**
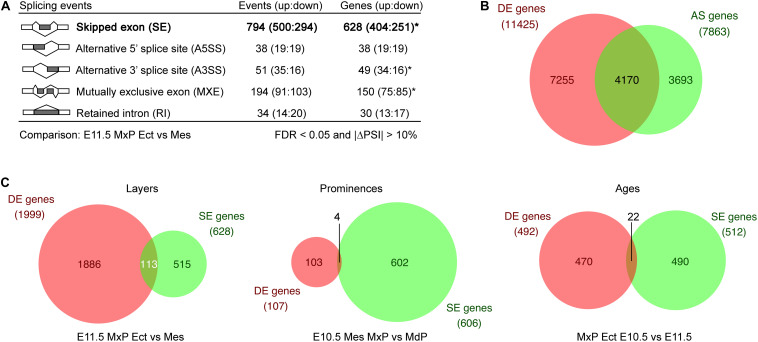
Alternative splicing program during facial development. **(A)** Alternative splicing events called by rMATS by categories from comparison between E11.5 MxP ectoderm and mesenchyme with cutoffs as FDR < 0.05, |ΔPSI| > 10%, and at least one sample with five inclusion read counts or skipping read counts. For the diagrams of the five types of alternative splicing events, dark gray rectangles are the alternative used exons or the retained intron, while white rectangles are the conserved exons. Lines indicate splicing. Asterisks indicate that some of the genes have both up events or down events. **(B)** Many genes are regulated only by differential alternative splicing during facial development. Venn diagram showing the comparison between differentially expressed (DE) genes and differentially used alternatively spliced (AS) genes during facial development across all tissues. **(C)** Venn diagrams of individual layer, prominence, or age comparisons demonstrate there are few overlaps between DE and SE genes. AS, alternative splicing; DE, differentially expressed; Ect, ectoderm; Mes, mesenchyme; MdP, mandibular prominence; MxP, maxillary prominence.

Importantly, though, the RNA-seq data extend previous analyses in several ways. First, E10.5 is fully represented here, by inclusion of three additional tissues (E10.5 MxP ectoderm, E10.5 MxP mesenchyme, E10.5 FNP ectoderm). Second, by generating a separate NE component from the developing FNP, the genetic programs of the early olfactory epithelium can be studied in isolation, and a more accurate picture of gene expression in the FNP mesenchyme can be determined (Supplementary Figure 3). Third, the wider dynamic range of RNA-seq identifies about 30% more differentially expressed genes across the various categories than the microarray data using the same cutoffs after normalizing sample size. Fourth, we can also compare the current data with the previous single cell RNA-seq dataset that concentrated on the E11.5 lambdoid junction region where the MxP and FNP regions come into close proximity prior to fusion (Li et al., 2019). Again, we find that there is strong agreement between the assignments in the RNA-seq and single cell RNA-seq datasets for genes associated with the olfactory epithelium, MxP or FNP mesenchyme, and ectoderm (Supplementary Figures 3B–D).

Based on the heatmap data shown in Figure 1B, we defined several broad programs of differential gene expression: early ectoderm, pan-ectoderm, NE, early and late mesenchyme, pan-mesenchyme, and a late class that appeared in all tissue layers. Functional annotation clustering of these categories (Supplementary Table 3) revealed that both the early ectoderm and mesenchyme had characteristics of growth, including terms related to mitosis and catabolism. In contrast, the late category had terms associated with ion channels, and the late mesenchyme grouping highlighted extracellular matrix development. These findings are consistent with a switch from growth to differentiation that occurs during the E10.5–E12.5 window. For the pan ectodermal category, terms indicative of cell:cell junctions, skin development and the Hippo signaling pathway were observed, whereas pan mesenchyme was enriched in terms associated with cell migration and angiogenesis. For the NE category, enriched terms reflected the development of olfactory neurons in this cell population. The results from the RNA-seq data extend and support the developmental programs noted in our previous microarray-based analysis of the facial transcriptome (Hooper et al., 2017).

### AS Program in Facial Developmen*t*

Another significant advantage of RNA-seq analysis is that differences in splicing can be detected from the sequences of the various transcripts. Therefore, to dissect the AS program during facial development, rMATS (Shen et al., 2014) was utilized to identify the frequency of different classes of differential splicing across all datasets. Initially, we used rMATS to compare splicing differences between the E11.5 MxP ectoderm and mesenchyme and this revealed the differential usage of all five categories of alternative splicing: skipped exon (SE), mutually exclusive exon, alternative 5′ splice site, alternative 3′ splice site, and retained intron between these samples (Figure 2A). Supplementary Figures 3E, 4 show examples of each of these categories from our datasets, and also reveals that the mutually exclusive exon assignment would be more accurately described as complex splicing events as previously noted (Wang and Rio, 2018). To gain a comprehensive understanding of the AS program during facial development, we next performed 44 more two-sample comparisons across layers, ages and prominences among the 20 conditions (Supplementary Figure 5). The significantly different (FDR < 0.05 and | ΔPSI| > 10%) AS events obtained from each comparison are summarized in Supplementary Table 4 and shown in Supplementary Data Sheet 1. Supplementary Table 5 displays AS events merged from all comparisons by AS category, providing a comprehensive overview of differentially used AS in the developing mouse face. These rMATS results demonstrate that SE is the dominant form of differentially used AS across most of the comparisons, particularly across layers, while retained intron, alternative 5′ splice sites and alternative 3′ splice sites were not frequently detected. More AS occurs across layers or ages than across prominences (Supplementary Table 4). The exception is that substantial AS occurs across the E10.5 prominences, particularly in the E10.5 mesenchyme, suggesting that a potential AS program helps define differences between the prominences at early times in facial development.

To determine if the AS program uses the same genes as the DE program during facial development, we compared DE genes (Supplementary Table 2) with AS genes detected by rMATS from the 45 comparisons (Supplementary Data Sheet 1). We found that ∼53% of the AS genes are also regulated at the level of DE during this period of facial development, whereas the remainder of AS genes are regulated only at the level of differential splicing (Figure 2B). Moreover, in single comparisons across layer, prominence, or age, the majority of genes showing differential SE usage are not regulated at the level of differential expression (Figure 2C). These results indicate that many genes are only regulated at the level of differential AS during facial development. Furthermore, they indicate that AS is a major regulator for the genetic programs acting within the developing face across time, layer, and prominence.

### SE Program During Facial Developmen*t*

**FIGURE 3:**
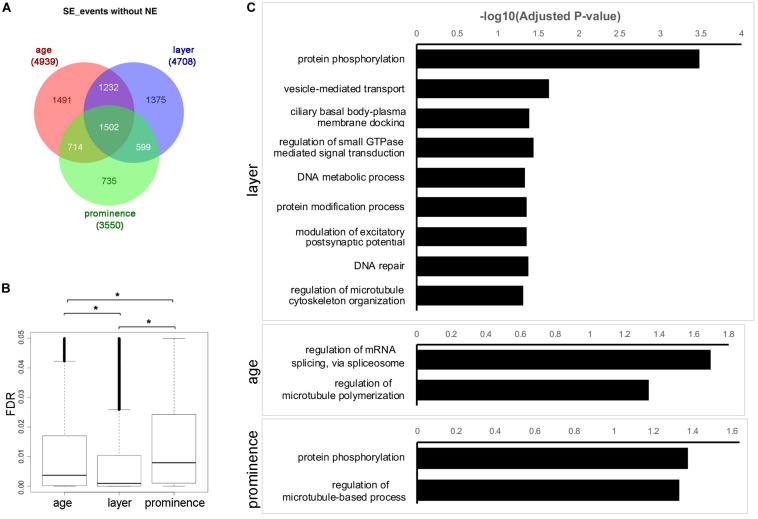
Skipped exon usage during facial development. **(A)** Venn diagram showing the differentially used SE events called across age, layer, and prominence comparisons, but omitting the nasal epithelium (NE) samples. The numbers of shared or unique SE events are marked in the corresponding area. **(B)** Boxplots of the FDR of differentially used SE events across age, layer, and prominence. *Significant with *t*-test. **(C)** GO Biological Process (GO_BP) term enrichment for genes associated with differentially used SE events across layer, age, and prominence with adjusted *p*-value < 0.05. Note that manual curation was used so that only the most significant term is shown in the bar charts for similar terms. The full list of enriched terms is available in Supplementary Table 6.

We next concentrated on the SE category, as this was the most prevalent AS event. To understand the SE program for facial formation, we aggregated the differentially used SE events across layer, age, and prominence (Supplementary Table 4 and Supplementary Data Sheet 1), with the exception of the NE samples which have their own unique expression profiles (Figure 1B). As shown in Figure 3A for the events, and in Supplementary Figure 6A for the corresponding genes, many SE events are differentially used by layer, age, and prominence during facial development. Meanwhile, about 1/5 to 1/3 SE events are uniquely used by layer, age or prominence, particularly for layer and age. This contrasts with DE for which the vast majority of genes show some degree of overlap within these categories (Figure 1C). In addition, the SE events of cross layer comparisons are the most statistically significant, followed by age comparisons and then prominence comparisons (Figure 3B).

To understand the biological functions of the genes with differential SE usage, GO annotations were retrieved from comparisons across age, layer or prominence (Figure 3C, Supplementary Figures 6B,C, and Supplementary Table 6). GO terms involved in cytoskeleton function (e.g., “microtubule binding”) and protein kinase activity were enriched in the SE events and corresponding genes for layer, age, and prominence comparisons as well as in the shared categories. Some terms were also specific to a particular comparison, such as “GTPase binding” for layer, and “pre-mRNA-binding” for age. We refined this analysis for layer by selecting only for events that occurred in all three prominences at a specific age. The individual layer event lists for all three ages were then combined together to generate a master list of > 300 genes that showed consistent differential AS between ectoderm and mesenchyme in all three prominences at one or more ages (Supplementary Table 7, “shared across layer” events). Similarly, for age, events were selected for a particular tissue layer only if they occurred in the ectoderm or mesenchyme of all three prominences between two time points. Subsequently the E10.5_vs_E11.5 and E11.5_vs_E12.5 event lists for ectoderm or mesenchyme were combined together to generate a list of ∼250 genes that showed consistent AS within the ectoderm or mesenchyme in all three prominences between one or two time points (Supplementary Table 7, “shared across age” events). Then, the genes associated with these core SE events for layer and age were annotated with Enrichr (Chen et al., 2013; Kuleshov et al., 2016) (Supplementary Figure 7 and Supplementary Table 7). As well as the previous terms noted in the individual comparisons, many additional terms involved in tissue establishment were enriched in the “shared across layer” events, such as establishment or maintenance of apical/basal polarity, myelination, and neuron projection development. With respect to KEGG annotations, Rap1 signaling and Hippo signaling pathways were also enriched in these “shared across layer” events. When the across-layer comparisons involving the NE samples were included, we found that these latter tissues were enriched for axonogenesis (Supplementary Table 8 and Supplementary Figure 8). In summary, these results indicate that layer, age, and prominence use both shared and unique AS strategies to shape facial development.

### Validation of SE Events Across Layer, Age and Prominence

**FIGURE 4:**
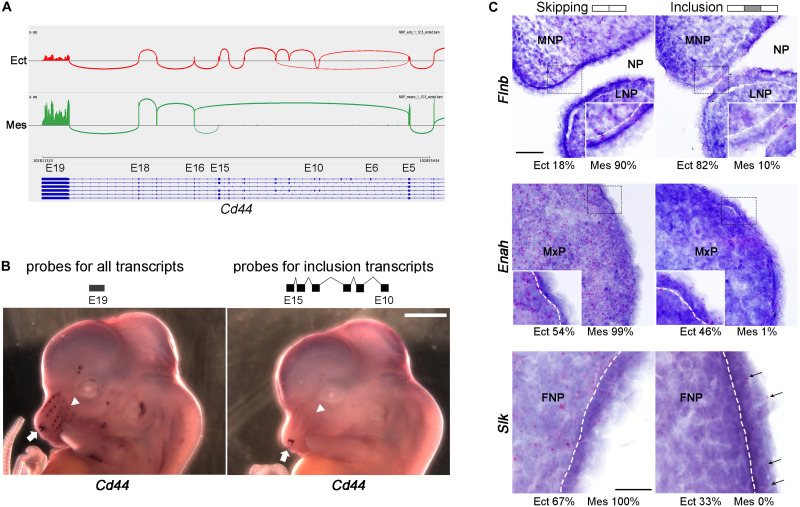
*In situ* hybridization validation of SE events across tissue layers. **(A)** Sashimi plot showing skipping of multiple exons (exons 6–15) of *Cd44* in mesenchyme. Sashimi plot was generated by using IGV browser with RNA-seq.bam files of E12.5 MxP ectoderm (Ect) sample 1 (top track, red) and E12.5 MxP mesenchyme (Mes) sample 1 (bottom track, green). The vertical piles are RNA-seq reads of exons, while the arcs connecting exons show how the exons are spliced. The isoform models are shown under the tracks with blue rectangles as exons, lines as introns, and arrowheads indicating the direction of transcripts (right to left). Exon (E) numbers of *Cd44* transcript variant 1 (NM_009851.2) are marked on top of the transcripts. Note that only the splicing junctions with five or more reads are shown here. **(B)** Whole mount *in situ* hybridization of E12.5 embryos for an exon 19 probe detecting all *Cd44* transcripts (left panel) or an exons 10–15 probe specific for ectodermal transcripts (right panel). The arrows point to the nasal cavity and the arrowheads point to vibrissae. Scale bar: 1 mm. **(C)** *In situ* hybridization on E11.5 frontal sections of FNP, MxP, and FNP using Basescope for SE events in *Flnb, Enah*, and *Slk*, respectively. Gene names are shown on the left and probes (skipping or inclusion) on top. The rMATS estimated average percentage of skipping and inclusion for Ect and Mes tissues (FNP for *Flnb*, MxP for *Enah*, and FNP for *Slk*) are shown under the corresponding panels. Red punctuate dots are signals for the Basescope probes. The sections are counterstained with hematoxylin. For all three genes the isoforms with the skipped exon are expressed in both ectoderm and mesenchyme, while the isoforms which include the alternative exon are mainly expressed in the ectoderm. Insets in *Flnb* and *Enah* panels show enlarged images of the areas in the dashed lined rectangles. White dashed lines indicate the boundary between ectoderm and mesenchyme. In the *Slk* panels, arrows indicate Basescope signals for the probe detecting the alternative exon. Scale bar is 50 μm for the *Flnb* and *Enah* panels, and scale bar for *Slk* is 20 μm. FNP, frontonasal process; LNP, lateral nasal process; MNP, medial nasal process; MxP, maxillary process; NP, nasal pit.

Several approaches were used for SE validation of the RNA-seq and rMATS data. The majority (75%) of the skipped exons detected were less than 160 nt in length and therefore difficult to detect using standard RNA *in situ* hybridization techniques. Moreover, the difference between tissue layer, prominence, and age was usually not absolute, but relative and so not readily distinguished using *in situ* hybridization. Therefore, we utilized different methodologies for validation based on the length of the skipped exons, and the ΔPSI. *Cd44*, a gene encoding multiple transmembrane protein isoforms involved in lymphocyte function and cancer metastasis (Baaten et al., 2010; Chen et al., 2018), was one of the most notable examples of a large difference in exon usage between tissue layers (Figure 4A and Supplementary Figure 9A). Using an *in situ* probe for the exons only present in the ectodermal transcripts, expression was detected mainly at the entrance to the nasal pit and in the tooth buds, with weaker expression associated with vibrissae (Figure 4B and Supplementary Figure 9). In contrast, a probe specific for a common exon that detects all isoforms showed more prominent staining of the developing vibrissae, as well as internal staining of the mesenchyme, confirming the prediction of rMATS. Moreover, the similar expression detected by the two types of *Cd44* probes in tooth buds and nasal pit is consistent with a previous study examining Cd44 isoform expression at later time points (Yu and Toole, 1997). For three genes with shorter SE events, *Flnb*, *Enah*, and *Slk*, we utilized the BaseScope^TM^ *in situ* hybridization technique. In each instance, Basescope^TM^ probes were designed to detect the splice junctions for either inclusion or skipping of an alternative exon (Supplementary Table 9 and Supplementary Figure 10). For all three genes, rMATS predicted that skipping mainly occurs in the mesenchyme, whereas the majority of inclusion is restricted to the ectoderm. Basescope^TM^ analysis confirmed these assignments, with probes for the skipping isoforms detecting expression in both ectoderm and mesenchyme of E11.5 facial prominences, while the inclusion isoforms were detected mainly in the ectoderm for all three genes (Figure 4C). Lastly, we used reverse transcription PCR to validate SE events that were called as differentially used across layer, age, and prominence (Supplementary Figures 11–13). Primers were designed to span the skipped exons (Supplementary Table 9), which amplified both the inclusion and skipped forms in one reaction generating PCR products of characteristic size. Even though this validation method is not quantitative, consistent changes were observed between the reverse transcription PCR results (8 events for layer, 11 events for age, and 5 for prominence) and the rMATS predictions. Notably, for genes including *Lef1*, *Lrrfip2*, *Numa1*, and *Phactr4*, we could detect changes in exon usage both over time and between tissue layers. Reverse transcription PCR was also able to validate more subtle differences in PSI than *in situ* hybridization. This was especially relevant to the rarer prominence differences in SE usage that occur for *Myo1b*, *Syne2*, *Exoc1*, and *Postn1*. In combination, these additional studies validated the differential SE usage occurring in the developing face discovered by bioinformatics analysis.

### Splicing Regulatory Program for Facial Developmen*t*

Given the changes in AS over time, between tissue layers and even between prominences, we next examined how the expression of both general and tissue-specific splicing factors correlated with these alterations. As a first step toward identifying genes that might drive the AS program, we profiled the differential expression of established RNA binding proteins (RBP) (Supplementary Figure 14 and Supplementary Table 10), defined as the mouse homologs of a comprehensive list of human RBPs (Gerstberger et al., 2014). Interestingly, a large group of RBPs were identified with high expression in the E10.5 samples, particularly in mesenchyme, that rapidly diminished at later time points. Many of these genes are required for ribosomal RNA processing, ribosomal biogenesis, and translation, e.g., *Rps29* (Supplementary Figures 14B,C and Supplementary Table 10). As noted above in reference to Figure 1 and Supplementary Table 3, these results support our contention that the facial gene expression program switches from growth to differentiation between E10.5–E12.5, as previously hypothesized (Feng et al., 2009; Hooper et al., 2017).

**FIGURE 5:**
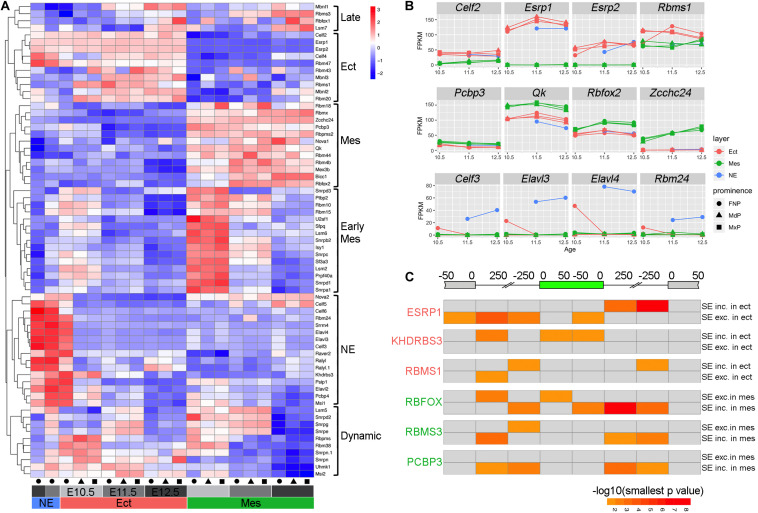
Regulatory program for alternative splicing during facial development. **(A)** Heatmap showing differentially expressed splicing factors during facial development (maximal FPKM across all samples > 1, adjusted *p*-value < 0.01, and maximal fold change across all samples > 2). Each row shows the scaled expression of a presumptive splicing regulator with the color scale at the top right, while each column represents average expression of a sample based on three biological replicates. The columns are annotated at the bottom with colors for tissue layers (red for Ect, green for Mes, and blue for NE), gray intensity for ages (light for E10.5, intermediate for E11.5, and dark for E12.5), and shapes for prominences (circle for FNP, triangle for MdP, and rectangle for MxP). The differentially expressed splicing factor programs are bracketed on the right side of the heatmap as late program in both ectoderm and mesenchyme (Late), pan ectoderm program (Ect), pan mesenchyme program (Mes), early mesenchyme program (Early Mes), nasal epithelial program (NE), and a program with complex dynamic change over time in ectoderm and mesenchyme (Dynamic). **(B)** Expression profiles for genes with higher expression in ectoderm (top panels), mesenchyme (middle panels), or nasal epithelium (bottom panels) during facial development. For each panel, the gene name is shown on the top, and the x and y axes show embryonic day and averaged FPKM from the three biological replicates for each sample, respectively. Layers are shown with colors (red for Ect, green for Mes, and blue for NE), while prominences are distinguished by shapes (circle for FNP, triangle for MdP, and rectangle for MxP). **(C)** Heatmap for RNA binding protein motifs on and around the skipped exon summarized from rMAP2 predictions of SE events from E11.5 MxP Ect_vs_Mes comparison (Supplementary Figure 15A). At the top, the diagram of the gene shows the skipped exon in green, the upstream and the downstream exons in gray. The sequences associated with the skipped exon are divided into eight regions according to the distance to the closest splicing junction. For each panel, the respective RBP is shown on the left. The top row is the significance [–log10(smallest *p*-value)] of the predicted RBP motif for each region in events with higher PSI in ectoderm/lower PSI in mesenchyme (SE inc. in ect/SE exc. in mes), and the bottom row is the significance of the predicted motif for each region in events with lower PSI in ectoderm/higher PSI in mesenchyme (SE exc. in ect/SE inc. in Mes). The color scale is shown in the bottom right, with darker color indicating more significant. The gray color indicates non-significant (smallest *p*-value > 0.05). Please note that in the same region, if it’s significant in both SE inc. in ect/SE exc. in mes and SE exc. in ect/SE inc. in Mes, only the more significant one is shown in the heatmap, while the less significant one is shown in gray. Ect, ectoderm; FNP, frontonasal process; Mes, mesenchyme; MdP, mandibular prominence; MxP, maxillary prominences; NE, nasal epithelium; SE, skipped exon.

Next, using a curated list of splicing regulators (Han et al., 2013; Bebee et al., 2015), we found that there was dynamic regulation of their mRNAs across layer and age and, to a lesser extent, prominence (Figures 5A,B and Supplementary Table 11). The NE, which at E10.5 is part of the FNP ectoderm sample, had a unique expression profile for these splicing factors, consistent with its distinctive expression and AS profile (Figure 1B, Supplementary Tables 2, 3, and Supplementary Data Sheet 1). Notable splicing factors expressed in the NE included Celf and Elavl family members as well as *Msi1* and *Srrm4* which are important for neurogenesis (Sakakibara et al., 1996; Irimia et al., 2014). Ectoderm-specific splicing factors included *Esrp1* and *Esrp2* that are critical regulators of ectodermal splicing for facial development (Warzecha et al., 2009; Bebee et al., 2015; Lee et al., 2020). Several splicing factors displayed higher relative expression in the early mesenchyme, including *Rbm10*, an AS factor mutated in human TARP Syndrome (Johnston et al., 2010). A number of other genes involved in AS showed specific pan- mesenchymal expression, such as *Rbfox2*, which is required in neural crest cells during craniofacial development (Cibi et al., 2019).

A number of these differentially expressed splicing regulators have defined binding sites that can be used in association with the rMAPS2 program (Hwang et al., 2020) to assess where these proteins bind with respect to differentially used exons. Therefore, to discover potential splicing regulators of the genes involved in facial development, we cross-referenced the binding motifs of the differentially expressed splicing regulators *Esrp*, *Rbms1, Khdrbs3, Rbms3, Rbfox*, and *Pcbp3* to the sequence within and around relevant skipped exons (Figure 5C and Supplementary Figure 15). In this context, previous studies with the ectodermal factor *Esrp1* have shown that its binding motif is highly enriched in the downstream intron of included exons in ectoderm tissues, but is also enriched in the upstream intron of the skipped exons (Bebee et al., 2015). Our analysis of differential SE events occurring between genes expressed in the E11.5 MxP ectoderm and mesenchyme support these findings and indicate that Esrp1 promotes inclusion of the exon when it binds to the downstream intron, while it represses the inclusion when it binds to the upstream intron (Figure 5C). The ectodermal factor Rbms1 binds within introns proximal to the 5′ side of both the included exon and the downstream exon when an exon is included, whereas for exon skipping it tends to bind within the 5′ intron closer to the upstream exon. For Khdrbs3, another ectodermal factor which is also a known splicing regulator for *Cd44* (Stoss et al., 2001), we determined that there was enrichment for the binding site in the upstream intron proximal to the upstream exon, as well as within the included exon itself. A corresponding set of predictions were also determined for the mesenchymal factors Rbfox, Rbms3, and Pcbp3 proteins with respect to differential exon skipping or inclusion in this tissue layer (Figure 5C). Rbfox sites were highly enriched in the downstream intron and slightly enriched in the 5′ intron and the body of the exon included in mesenchymal tissue. Meanwhile, there was slight enrichment of Rbfox sites in some regions of the exon skipped in mesenchymal tissue. However, in contrast to Rbfox, Pcbp3 sites were only enriched around the exon included in mesenchymal tissue, but did not show any enrichment around the exons skipped in mesenchymal tissues. In combination, these data show different strategies involved in differential splicing of exons by these RNA binding proteins, and reveal that a complex and integrated program of AS occurs during a critical phase of craniofacial development.

## Discussion

The formation of the mammalian face is a complex process involving growth, morphogenesis, and fusion. In this respect, development of the human face is among the most sensitive systems affected by both genetic and environmental perturbations. To understand the gene regulatory programs underlying facial development, we have performed RNA-seq analysis during a critical period of mouse face development. Specifically, we have investigated gene expression within the ectoderm and mesenchyme of each of the three facial prominences at three stages, E10.5, E11.5, and E12.5. This period begins when all three prominences are distinct entities until their fusion to create the lower and upper jaws, and the primary palate. These efforts were part of the larger NIDCR FaceBase 2 consortium, and they extend and complement other information available through FaceBase as well as additional gene expression analyses (Gong et al., 2005; Feng et al., 2009; Brunskill et al., 2014; Brinkley et al., 2016; Hooper et al., 2017; Li et al., 2019). These studies are also relevant to the early separation and development of the olfactory pit and specialized olfactory epithelium that are derived from the frontonasal prominence.

Examination of differential expression across these 60 samples revealed specific ectodermal and mesenchymal gene expression programs that change over time. Importantly, over the 48 h period under analysis, there was a general switch from genes and ontologies associated with growth, mitosis, and catabolism to those associated with differentiation, extracellular matrix development, and the formation of cell:cell junctions. There was also a major reduction in RBPs associated with rRNA processing and ribosomal biogenesis between E10.5 and E12.5 correlating with the aforementioned changes in growth. However, other groups of RBPs involved in splicing, RNA export, or mRNA stability showed more complex expression profiles during this period. There were two main reasons we were especially interested in the expression of such RBPs. First, in this study we were particularly focused on the extent and regulation of differential splicing in our dataset. Second, the importance of such RBPs in facial development has been revealed by human genetics. Thus, several genes associated with ribosomal biogenesis, including some associated with pre-rRNA processing, cause complex pathology often including craniofacial defects – such as the mutation of *TCOF1* in Treacher Collins Syndrome and the mutation of ribosomal protein genes in Diamond Blackfan Anemia (Farley-Barnes et al., 2019). Aside from these ribosomopathies, changes in RBPs associated with mRNA stability, transport, or splicing are also linked with craniofacial defects. Thus, in humans, mutations in *SNRPB*, *TXNL4A*, *EIF4A3*, *SF3B4*, *HNRNPH2*, *PUF60*, *FAM172A*, *EFTUD2*, *CWC27*, and *RBM10* cause various syndromes that have a craniofacial component in their pathology (Bain et al., 2016; Marques et al., 2016; Loiselle and Sutherland, 2018; Berube-Simard and Pilon, 2019; Beauchamp et al., 2020; Griffin and Saint-Jeannet, 2020). In some cases, these factors are involved in general spliceosome function, for example *SF3B4*, which is mutated in Nager Syndrome. In other instances, such RBPs are involved in AS, such as *RBM10* which is mutated in TARP syndrome (Sutherland et al., 2017). These findings indicate the importance of both general splicing and AS in multiple processes including development of the heart, eye, nervous system, limb, and face (Sutherland et al., 2017; Loiselle and Sutherland, 2018; Griffin and Saint-Jeannet, 2020; Yamada et al., 2020). The datasets we have generated can be mined to determine how expression of such human genes occurs in the developing mouse face with respect to age, prominence, and layer and how their alteration might impact normal craniofacial development.

Studies in mouse have also demonstrated the critical importance of splicing factors for craniofacial development. Notably, loss of *Esrp1*, an ectodermal specific factor involved in the differential splicing of a number of critical targets including Fgf Receptors, results in cleft lip/palate (Bebee et al., 2015; Lee et al., 2020). This phenotype is further exacerbated by the additional deletion of the related *Esrp2* gene (Bebee et al., 2015). Interestingly, a role for the Esrp family in craniofacial development is also conserved in zebrafish, in which the genes regulate ethmoid plate development, with these proteins acting on a core collection of exon targets between human, mouse and zebrafish (Burguera et al., 2017). Second, within the neural crest derived mesenchyme, deletion of the alternative splicing factor *Rbfox2* results in cleft secondary palate (Cibi et al., 2019). Third, a hypomorphic mutation in *Fam172a* – linked to co-transcriptional AS – was also found to result in cleft secondary palate as well as a lower incidence of other craniofacial defects, mimicking certain aspects of CHARGE syndrome caused by human *FAM172A* alterations (Belanger et al., 2018). However, it is important to consider that alterations in sequences between species, which may not change coding potential or constitutive splicing, may still change the *cis*-acting sequences necessary for binding an RBP involved in alternative splicing. Therefore, mutation of a splicing factor may not always impact the same targets in different species, potentially leading to different developmental outcomes (Burguera et al., 2017). A similar potential species-specific difference in overall phenotype might also be expected for spliceosomopathies given the variation in the number of genes that can spliced between various species, as well as differences in epigenetic chromatin marks, and strength of splice site (Barbosa-Morais et al., 2012; Merkin et al., 2012; Baralle and Giudice, 2017; Yamada et al., 2020).

These studies in humans and mice demonstrate the critical importance of both constitutive as well as alternative splicing factors in craniofacial development. However, we only have a limited understanding of the extent of AS during facial development, and how it is regulated within the individual facial prominences, across tissue layers, and particularly with developmental age. Here, the sequencing depth we have performed for all 60 RNA-Seq samples provided the opportunity to analyze both differential expression and local differences in differential splicing of transcripts. One notable observation was that the number of genes affected by AS across these datasets is in the same range as genes altered by differential expression. Many of these AS genes are not regulated at the transcriptional level in our comparisons suggesting that AS acts as a significant component of the regulatory network guiding facial development. AS differences for tissue layer and age were the most prevalent, while those distinguishing the facial prominences were more minor. Layer comparisons highlighted genes involved in regulation of small GTPase mediated signal transduction, such as ARHGAPs and ARHGEFs, as well as those involved in apical basal polarity and Hippo signaling (Supplementary Tables 6, 7). Layer specific differences correlated with the expression of different splicing factors within these tissues, notably *Esrp1* and *Esrp2* in the ectoderm, and *Qk* and *Rbfox2* in the mesenchyme. As noted above, tissue-specific removal of either *Esrp1* or *Rbfox2* also results in mouse craniofacial defects, and further studies on these mutant mouse models revealed AS disruptions of multiple target genes (Belanger et al., 2018; Cibi et al., 2019; Lee et al., 2020). Significantly, loss of *Esrp1* caused a switch for multiple genes from an ectodermal pattern of AS toward a mesenchymal pattern, typified by a switch of *Fgfr2* to an isoform with greater inclusion of the III-c exon (Lee et al., 2020). Conversely, loss of *Rbfox2* switched splicing from a mesenchymal to an ectodermal pattern, as seen for AS of *Map3k7* (Cibi et al., 2019). However, such changes are often not absolute, presumably because these AS events are coordinately regulated by multiple factors. Together, though, the studies on Esrp1 and Rbfox2 protein function reveal the central importance of such tissue-specific splicing regulators for facial development. With respect to our studies on layer specific AS, Lee et al. (2020) also examined layer specific AS differences at a single time point within the whole face using RNA-seq. Although these two analyses are not identical, there was still significant agreement between them strengthening our conclusions. In this respect, we detected equivalent changes between ectoderm and mesenchyme for the six genes validated by PCR in Lee et al. (2020) – *Arhgef10l*, *Epb41*, *Lsm14b*, *Magi1*, *Myo1b*, and *Usp4* (Supplementary Table 5). Further, both studies highlighted that skipped exons were the most common AS event, and detected similar layer-specific ontologies including “Adherens Junctions” and “MAPK signaling pathway” (Lee et al., 2020). Also, with respect to layer, as the nasal pit and olfactory epithelium form, there are both splicing changes and concomitant splicing factor changes – such as the induction of Celf and Elavl factor expression – that correlate with the onset of neurogenesis.

Age related changes in AS correlated with both increased and decreased expression of certain RBPs over time. Some RBPs, including *Ptbp2* and *Rbm10*, showed reduced expression between E10.5 and E12.5 in all tissue layers and prominences. Conversely, expression of splicing factors including *Mbnl1*, *Mbnl2*, and *Zcchc24* increased during this developmental window. These observations support previous tissue culture studies showing that these three splicing factors rise in expression during the transition between stem cells and more differentiated cell types (Han et al., 2013; Cieply et al., 2016). Moreover, these previous studies identified several potential targets of *Zcchc24 – Spag9 –* and the Mbnl proteins – *Ssbp3*, *Exoc1*, *Macf1*, *Tead1*, and *Mta1* – and we see concomitant changes in AS of these transcripts over time that correlate with the increased expression of these three RBPs (Supplementary Table 5).

In terms of ontogeny, age related AS changes highlighted RNA binding associated regulatory processes. This observation suggests a network of splicing cascades, autoregulation of splicing factors, or cross-regulation of other RBPs involved in RNA stability, transport, or translation that occurs throughout the E10.5–E12.5 period. Such networks, promoting differentiation, lineage choices and tissue homeostasis are well-described in other physiological and developmental systems (Jangi and Sharp, 2014). For example, the splicing factor Ptbp1 causes nonsense mediated decay of *Ptbp2* via alternative splicing and this regulatory switch controls neuronal differentiation (Jangi and Sharp, 2014). Notably, the splicing factors *Ptbp1* and *Ptbp2* are expressed in all facial tissues and prominences throughout the E10.5–E12.5 window (Figure 5A and Supplementary Table 11), raising the possibility that this regulatory circuit also operates during facial development. Microtubule binding was another significant age-related term, and a number of the associated AS genes in this category have functions in mitosis and centrosome function. Studies in human cell lines have examined the occurrence of AS across the cell cycle, many coordinately controlled by CLK1, a kinase that regulates several proteins involved in splicing (Dominguez et al., 2016). *Clk1* exhibits both DE and AS in our dataset (Supplementary Tables 2, 5) and we also noted that three of its targets – *Mdm1*, *Clasp1*, and *Cdk5rap2 –* showed consistent AS differences over age in the mouse face (Supplementary Table 5). The inclusion or exclusion of particular exons is believed to have several broad effects on protein function (Ule and Blencowe, 2019). In certain instances, these changes can introduce an in-frame stop codon which will cause a loss of functional protein expression, as described above for *Ptbp2*. Alternatively, a more disordered region may be introduced into a protein that acts as the site of regulation through phosphorylation. In the context of *Mdm1*, an additional exon is incorporated over time even as overall message levels drop for this gene (Supplementary Tables 2, 5). Notably, the new exon encodes an interaction site for Rho kinase regulation, suggesting a link between age-dependent splicing, cell cycle regulation, and the cytoskeleton (Chang et al., 2008). However, the consequences of many AS changes we observe on protein function remain to be elucidated. Here, isoform specific gene manipulation has the potential to reveal the consequences of AS on protein function, a prime example being the Fgfr2 IIIb and IIIc mouse knockout models which have significant relevance to craniofacial development (De Moerlooze et al., 2000; Hajihosseini et al., 2001). However, unexpected splicing outcomes caused by loss of an exon can complicate a straightforward analysis of the expected isoform changes (Möröy and Heyd, 2007). Nevertheless, the rapid advances in splice switching oligonucleotide therapy for human conditions including spinal muscular atrophy raise the possibility of treating craniofacial conditions resulting from aberrant splicing (Scotti and Swanson, 2016).

In summary, the current analysis provides important insight into differential gene expression and AS with respect to age, layer and prominence during facial development, details correlations between AS and RBP gene expression, and helps reveal how potential splicing factor recognition motifs map in the vicinity of skipped exons to regulate AS. When coupled with studies determining how loss of splicing factors can alter AS splicing patterns during facial development (Belanger et al., 2018; Cibi et al., 2019; Lee et al., 2020), our findings reveal the widespread occurrence and importance of splicing differences during facial development and will hopefully act as a springboard for further experimental analysis of how this important regulatory process shapes the mammalian face.

## Data Availability Statement

All data are available at the FaceBase website under the accession number FB00000867 (https://www.facebase.org/chaise/record/#1/isa:dataset/RID=TJA).

## Ethics Statement

The animal study was reviewed and approved by University of Colorado Anschutz Medical Campus Animal Care and Usage Committee.

## Author Contributions

TW and JH conceived the RNA-seq analysis and alternative splicing analysis, respectively, with assistance from KJ who also performed the initial RNA-seq analysis. HL was responsible for acquisition of biological samples, as well as differential expression and alternative splicing analyses. HL, TW, and FS contributed to data validation. HL and TW wrote the manuscript. All authors contributed to manuscript revision and approved the submitted version.

## Conflict of Interest

The authors declare that the research was conducted in the absence of any commercial or financial relationships that could be construed as a potential conflict of interest.

## Footnotes

1 https://www.facebase.org/

2 http://research-pub.gene.com/gmap/

3 http://cole-trapnell-lab.github.io/cufflinks/

4 http://rnaseq-mats.sourceforge.net

5 https://amp.pharm.mssm.edu/Enrichr/

## References

[B1] Baaten B. J. Li C. R. Bradley L. M. (2010). Multifaceted regulation of T cells by CD44. *Commun Integr Biol.* 3 508–512. 10.4161/cib.3.6.13495 21331226 PMC3038050

[B2] Bain J. M. Cho M. T. Telegrafi A. Wilson A. Brooks S. Botti C. (2016). Variants in HNRNPH2 on the X chromosome are associated with a neurodevelopmental disorder in females. *Am. J. Hum. Genet.* 99 728–734. 10.1016/j.ajhg.2016.06.028 27545675 PMC5011042

[B3] Baralle F. E. Giudice J. (2017). Alternative splicing as a regulator of development and tissue identity. *Nat. Rev. Mol. Cell Biol.* 18 437–451. 10.1038/nrm.2017.27 28488700 PMC6839889

[B4] Barbosa-Morais N. L. Irimia M. Pan Q. Xiong H. Y. Gueroussov S. Lee L. J. (2012). The evolutionary landscape of alternative splicing in vertebrate species. *Science* 338 1587–1593. 10.1126/science.1230612 23258890

[B5] Beauchamp M. C. Alam S. S. Kumar S. Jerome-Majewska L. A. (2020). Spliceosomopathies and neurocristopathies: two sides of the same coin?. *Dev. Dyn.* 249 924–945. 10.1002/dvdy.183 32315467

[B6] Bebee T. W. Park J. W. Sheridan K. I. Warzecha C. C. Cieply B. W. Rohacek A. M. (2015). The splicing regulators Esrp1 and Esrp2 direct an epithelial splicing program essential for mammalian development. *eLife* 4:e08954. 10.7554/eLife.08954 PMC4566030 26371508

[B7] Belanger C. Berube-Simard F. A. Leduc E. Bernas G. Campeau P. M. Lalani S. R. (2018). Dysregulation of cotranscriptional alternative splicing underlies CHARGE syndrome. *Proc. Natl. Acad. Sci. U.S.A.* 115 E620–E629. 29311329 10.1073/pnas.1715378115 PMC5789929

[B8] Berube-Simard F. A. Pilon N. (2019). Molecular dissection of charge syndrome highlights the vulnerability of neural crest cells to problems with alternative splicing and other transcription-related processes. *Transcription* 10 21–28. 10.1080/21541264.2018.1521213 30205741 PMC6351119

[B9] Brinkley J. F. Fisher S. Harris M. P. Holmes G. Hooper J. E. Jabs E. W. (2016). The FaceBase consortium: a comprehensive resource for craniofacial researchers. *Development* 143 2677–2688. 10.1242/dev.135434 27287806 PMC4958338

[B10] Brunskill E. W. Potter A. S. Distasio A. Dexheimer P. Plassard A. Aronow B. J. (2014). A gene expression atlas of early craniofacial development. *Dev. Biol.* 391 133–146. 10.1016/j.ydbio.2014.04.016 24780627 PMC4095820

[B11] Burguera D. Marquez Y. Racioppi C. Permanyer J. Torres-Méndez A. Esposito R. (2017). Evolutionary recruitment of flexible Esrp-dependent splicing programs into diverse embryonic morphogenetic processes. *Nat. Commun.* 8:1799. 10.1038/s41467-017-01961-y PMC5703972 29180615

[B12] Chai Y. Maxson R. E. Jr. (2006). Recent advances in craniofacial morphogenesis. *Dev. Dyn.* 235 2353–2375. 10.1002/dvdy.20833 16680722

[B13] Chang B. Mandal M. N. Chavali V. R. Hawes N. L. Khan N. W. Hurd R. E. (2008). Age-related retinal degeneration (arrd2) in a novel mouse model due to a nonsense mutation in the Mdm1 gene. *Hum. Mol. Genet.* 17 3929–3941. 10.1093/hmg/ddn295 18805803 PMC2638579

[B14] Chen C. Zhao S. Karnad A. Freeman J. W. (2018). The biology and role of CD44 in cancer progression: therapeutic implications. *J. Hematol. Oncol.* 11:64. 10.1186/s13045-018-0605-5 PMC5946470 29747682

[B15] Chen E. Y. Tan C. M. Kou Y. Duan Q. Wang Z. Meirelles G. V. (2013). Enrichr: interactive and collaborative HTML5 gene list enrichment analysis tool. *BMC Bioinformatics* 14:128. 10.1186/1471-2105-14-128 23586463 PMC3637064

[B16] Cibi D. M. Mia M. M. Guna Shekeran S. Yun L. S. Sandireddy R. Gupta P. (2019). Neural crest-specific deletion of Rbfox2 in mice leads to craniofacial abnormalities including cleft palate. *eLife* 8:e45418. 10.7554/eLife.45418 PMC6663295 31241461

[B17] Cieply B. Park J. W. Nakauka-Ddamba A. Bebee T. W. Guo Y. Shang X. (2016). Multiphasic and dynamic changes in alternative splicing during induction of pluripotency are coordinated by numerous RNA-binding proteins. *Cell Rep.* 15 247–255. 10.1016/j.celrep.2016.03.025 27050523 PMC5718363

[B18] De Moerlooze L. Spencer-Dene B. Revest J. M. Hajihosseini M. Rosewell I. Dickson C. (2000). An important role for the IIIb isoform of fibroblast growth factor receptor 2 (FGFR2) in mesenchymal-epithelial signalling during mouse organogenesis. *Development* 127 483–492. 10631169 10.1242/dev.127.3.483

[B19] Dixon M. J. Marazita M. L. Beaty T. H. Murray J. C. (2011). Cleft lip and palate: understanding genetic and environmental influences. *Nat. Rev. Genet.* 12 167–178. 10.1038/nrg2933 21331089 PMC3086810

[B20] Dominguez D. Tsai Y. H. Weatheritt R. Wang Y. Blencowe B. J. Wang Z. (2016). An extensive program of periodic alternative splicing linked to cell cycle progression. *eLife* 5:e10288. 10.7554/eLife.10288 PMC4884079 27015110

[B21] Farley-Barnes K. I. Ogawa L. M. Baserga S. J. (2019). Ribosomopathies: old concepts, new controversies. *Trends Genet.* 35 754–767. 10.1016/j.tig.2019.07.004 31376929 PMC6852887

[B22] Feng W. Leach S. M. Tipney H. Phang T. Geraci M. Spritz R. A. (2009). Spatial and temporal analysis of gene expression during growth and fusion of the mouse facial prominences. *PLoS One* 4:e8066. 10.1371/journal.pone.0008066 20016822 PMC2789411

[B23] Gerstberger S. Hafner M. Tuschl T. (2014). A census of human RNA-binding proteins. *Nat. Rev. Genet.* 15 829–845. 10.1038/nrg3813 25365966 PMC11148870

[B24] Gong S. G. Gong T. W. Shum L. (2005). Identification of markers of the midface. *J. Dent. Res.* 84 69–72. 10.1177/154405910508400112 15615879

[B25] Griffin C. Saint-Jeannet J. P. (2020). Spliceosomopathies: diseases and mechanisms. *Dev. Dyn.* [Epub ahead of print]. 10.1002/dvdy.214 32506634 PMC8603363

[B26] Hajihosseini M. K. Wilson S. De Moerlooze L. Dickson C. (2001). A splicing switch and gain-of-function mutation in FgfR2-IIIc hemizygotes causes Apert/Pfeiffer-syndrome-like phenotypes. *Proc. Natl. Acad. Sci. U.S.A.* 98 3855–3860. 10.1073/pnas.071586898 11274405 PMC31142

[B27] Han H. Irimia M. Ross P. J. Sung H. K. Alipanahi B. David L. (2013). MBNL proteins repress ES-cell-specific alternative splicing and reprogramming. *Nature* 498 241–245. 10.1038/nature12270 23739326 PMC3933998

[B28] Hooper J. E. Feng W. Li H. Leach S. M. Phang T. Siska C. (2017). Systems biology of facial development: contributions of ectoderm and mesenchyme. *Dev. Biol.* 426 97–114. 10.1016/j.ydbio.2017.03.025 28363736 PMC5530582

[B29] Hwang J. Y. Jung S. Kook T. L. Rouchka E. C. Bok J. Park J. W. (2020). rMAPS2: an update of the RNA map analysis and plotting server for alternative splicing regulation. *Nucleic Acids Res.* 48 W300–W306. 32286627 10.1093/nar/gkaa237 PMC7319468

[B30] Irimia M. Weatheritt R. J. Ellis J. D. Parikshak N. N. Gonatopoulos-Pournatzis T. Babor M. (2014). A highly conserved program of neuronal microexons is misregulated in autistic brains. *Cell* 159 1511–1523. 10.1016/j.cell.2014.11.035 25525873 PMC4390143

[B31] Jangi M. Sharp P. A. (2014). Building robust transcriptomes with master splicing factors. *Cell* 159 487–498. 10.1016/j.cell.2014.09.054 25417102 PMC4243530

[B32] Jiang R. Bush J. O. Lidral A. C. (2006). Development of the upper lip: morphogenetic and molecular mechanisms. *Dev. Dyn.* 235 1152–1166. 10.1002/dvdy.20646 16292776 PMC2562450

[B33] Johnston J. J. Teer J. K. Cherukuri P. F. Hansen N. F. Loftus S. K. Chong K. (2010). Massively parallel sequencing of exons on the X chromosome identifies RBM10 as the gene that causes a syndromic form of cleft palate. *Am. J. Hum. Genet.* 86 743–748. 10.1016/j.ajhg.2010.04.007 20451169 PMC2868995

[B34] Kuleshov M. V. Jones M. R. Rouillard A. D. Fernandez N. F. Duan Q. Wang Z. (2016). Enrichr: a comprehensive gene set enrichment analysis web server 2016 update. *Nucleic Acids Res.* 44 W90–W97. 27141961 10.1093/nar/gkw377 PMC4987924

[B35] Lee S. Sears M. J. Zhang Z. Li H. Salhab I. Krebs P. (2020). Cleft lip and cleft palate in Esrp1 knockout mice is associated with alterations in epithelial-mesenchymal crosstalk. *Development* 147:dev187369. 10.1242/dev.187369 32253237 PMC7225129

[B36] Li H. Jones K. L. Hooper J. E. Williams T. (2019). The molecular anatomy of mammalian upper lip and primary palate fusion at single cell resolution. *Development* 146:dev174888. 10.1242/dev.174888 31118233 PMC6602358

[B37] Li H. Williams T. (2013). Separation of mouse embryonic facial ectoderm and mesenchyme. *J. Vis. Exp.* 74:50248. 10.3791/50248 PMC3654862 23603693

[B38] Loiselle J. J. Sutherland L. C. (2018). RBM10: harmful or helpful-many factors to consider. *J. Cell. Biochem.* 119 3809–3818. 10.1002/jcb.26644 29274279 PMC5901003

[B39] Marques F. Tenney J. Duran I. Martin J. Nevarez L. Pogue R. (2016). Altered mrna splicing, chondrocyte gene expression and abnormal skeletal development due to SF3B4 mutations in Rodriguez Acrofacial Dysostosis. *PLoS Genet.* 12:e1006307. 10.1371/journal.pgen.1006307 27622494 PMC5021280

[B40] Merkin J. Russell C. Chen P. Burge C. B. (2012). Evolutionary dynamics of gene and isoform regulation in mammalian tissues. *Science* 338 1593–1599. 10.1126/science.1228186 23258891 PMC3568499

[B41] Möröy T. Heyd F. (2007). The impact of alternative splicing in vivo: mouse models show the way. *RNA* 13 1155–1171. 10.1261/rna.554607 17563071 PMC1924907

[B42] Pan Q. Shai O. Lee L. J. Frey B. J. Blencowe B. J. (2008). Deep surveying of alternative splicing complexity in the human transcriptome by high-throughput sequencing. *Nat. Genet.* 40 1413–1415. 10.1038/ng.259 18978789

[B43] Sakakibara S. Imai T. Hamaguchi K. Okabe M. Aruga J. Nakajima K. (1996). Mouse-Musashi-1, a neural RNA-binding protein highly enriched in the mammalian CNS stem cell. *Dev. Biol.* 176 230–242. 10.1006/dbio.1996.0130 8660864

[B44] Samuels B. D. Aho R. Brinkley J. F. Bugacov A. Feingold E. Fisher S. (in press). FaceBase 3: analytical tools and FAIR resources for craniofacial and dental research. 10.1006/dbio.1996.0130 32958507 PMC7522026

[B45] Scotti M. M. Swanson M. S. (2016). RNA mis-splicing in disease. *Nat. Rev. Genet.* 17 19–32. 10.1038/nrg.2015.3 26593421 PMC5993438

[B46] Shen S. Park J. W. Lu Z. X. Lin L. Henry M. D. Wu Y. N. (2014). rMATS: robust and flexible detection of differential alternative splicing from replicate RNA-seq data. *Proc. Natl. Acad. Sci. U.S.A.* 111 E5593–E5601. 25480548 10.1073/pnas.1419161111 PMC4280593

[B47] Stoss O. Olbrich M. Hartmann A. M. Konig H. Memmott J. Andreadis A. (2001). The STAR/GSG family protein rSLM-2 regulates the selection of alternative splice sites. *J. Biol. Chem.* 276 8665–8673. 10.1074/jbc.m006851200 11118435

[B48] Sutherland L. C. Thibault P. Durand M. Lapointe E. Knee J. M. Beauvais A. (2017). Splicing arrays reveal novel RBM10 targets, including SMN2 pre-mRNA. *BMC Mol. Biol.* 18:19. 10.1186/s12867-017-0096-x 28728573 PMC5520337

[B49] Ule J. Blencowe B. J. (2019). Alternative splicing regulatory networks: functions, mechanisms, and evolution. *Mol. Cell.* 76 329–345. 10.1016/j.molcel.2019.09.017 31626751

[B50] Wang E. T. Sandberg R. Luo S. Khrebtukova I. Zhang L. Mayr C. (2008). Alternative isoform regulation in human tissue transcriptomes. *Nature* 456 470–476. 10.1038/nature07509 18978772 PMC2593745

[B51] Wang Q. Rio D. C. (2018). JUM is a computational method for comprehensive annotation-free analysis of alternative pre-mRNA splicing patterns. *Proc. Natl. Acad. Sci. U.S.A.* 115 E8181–E8190. 30104386 10.1073/pnas.1806018115 PMC6126775

[B52] Warzecha C. C. Sato T. K. Nabet B. Hogenesch J. B. Carstens R. P. (2009). ESRP1 and ESRP2 are epithelial cell-type-specific regulators of FGFR2 splicing. *Mol. Cell.* 33 591–601. 10.1016/j.molcel.2009.01.025 19285943 PMC2702247

[B53] Wrenn J. T. Wessells N. K. (1984). The early development of mystacial vibrissae in the mouse. *J. Embryol. Exp. Morphol.* 83 137–156. 6502072

[B54] Yamada T. Takechi M. Yokoyama N. Hiraoka Y. Ishikubo H. Usami T. (2020). Heterozygous mutation of the splicing factor Sf3b4 affects development of the axial skeleton and forebrain in mouse. *Dev. Dyn.* 249 622–635. 10.1002/dvdy.148 31900962

[B55] Yu Q. Toole B. P. (1997). Common pattern of CD44 isoforms is expressed in morphogenetically active epithelia. *Dev. Dyn.* 208 1–10. 10.1002/(sici)1097-0177(199701)208:1<1::aid-aja1>3.0.co;2-m 8989516

