# Supplementary material for: An Alternative Splicing Program for Mouse Craniofacial Development
Source: Front Physiol. 2020 Sep 4;11:1099. doi: 10.3389/fphys.2020.01099 (PMC7498679; doi:10.3389/fphys.2020.01099)
Supplement: Supplementary file 9 [file Table_9.pdf]

Supplementary Table 9. Primers and probes used for validation

A. Primers for making isoform specific probes.

Probes	Forward primer	Reverse primer	Probe size
Cd44 Ect	GGCTGATATAGACAGAATC AGCACCAGTGCTCATGGAG	CGTTGGAGTCAGTAGCAAGA GTCACTTCAGTTTCTCC	~700 nts
Cd44 TOT	GTACCGGTCCATCTTCCAT CTTGACCCGTTGTGC	GGAAGATCCAGGAGCAATG GGACATCCTGGCAGATG	~730 nts

B. Basescope probes were custom ordered from Advanced Cell Diagnostics for detecting skipping or inclusion of small exons. The catalog number (Cat No.) and the type of differential exon usage for the Basescope probes are as follows.

Gene	Probe name	Cat No.	Detecting isoforms
Flnb	BaseScope™ Probe- BA-Mm-Flnb-tvX1-E29E30	709401	Skipping
	BaseScope™ Probe- BA-Mm-Flnb-tv2-E30E31	709391	Inclusion
Enah	BaseScope™ Probe- BA-Mm-Enah-tv1-E12E13	709371	Skipping
	BaseScope™ Probe- BA-Mm-Enah-tvX2-E13E14	709361	Inclusion
Slk	BaseScope™ Probe- BA-Mm-Slk-tv2-E12E13	709431	Skipping
	BaseScope™ Probe- BA-Mm-Slk-tv1-E13E14	709421	Inclusion

C. Primers for RT-PCR validation:

Gene	Forward primer	Reverse primer	PCR product sizes
Lef1	AGCTCCTGAAATCCC CACCTTCTAC	TGGATGAGGGATG CCAGTTGTGTGG	126 bp, 217 bp
Evi5	AATCCAGTCAGCTTA CCAAGTC	GGCTTCACTCAGT CTGGCTT	242 bp, 278bp, 426 bp

Lrrfip2	ACAACGCCTCTAAGTGGGAA	CTGCACATGTGTTTCTGCCT	290 bp, 362 bp
Numa1	CAAGTACGAAGGTGCCAAGG	CTTGTAATGCTCAGCTGCCT	262 bp, 304 bp
Ocrl	TCGAGAAGTTCCTGTCACCA	TCTGCTGTAACTCCTCCTGC	177 bp, 221 bp
Map2	CTGCCGGACCTGAAGAATGT	GGTACGTGGTGAGCATTGTC	262 bp, 344 bp
*Phactr4**	GCAGTGATAAGTTCAAAGAGACA	GCTTCCAGTTGAGCCAAGTC	203 bp, 278bp*, 284 bp
Plod2	TGGGTACTATGCTCGCTCTG	GCAGTTGATATCAGCCGTCC	252 bp, 315 bp
Gpr137	AAGCGTCGGCCAGAGATGAG	GCTGCCAGGTTGTAACAGGC	111 bp, 337 bp
Myo1b	GAAGCATCAGAAGCGCTGTA	ACAGCAACTGCATGCTTACG	123 bp, 210 bp
Slain2	ATACCTCGAATGCAGCCTCAG	GTGAGGAGGTGGTTTGTGTGA	169 bp, 247 bp
Sorbs1	CAGCTGATTACTTGGAGTCC	TGTAGATGTAAACAACGTCTCC	157 bp, 315 bp
Mta1	GCAGCACAATGGGGTAGATG	GTCGATCCAGTTCATTCGCC	163 bp, 199 bp
Scarb1	CTGATGCCCCAGGTTCTTCA	GGCTTATAGTGTCTTCAGGAC	118 bp, 247 bp
Postn exon 17	TTTGTTCGTGGCAGCACCTTC	CTCCGTGGATCACTTCTGTCA	136 bp, 217 bp
Postn exon 21	ACAGGAGGTGGAGAAACAGG	GTCAGTGTGGTGGCTCTTACA	215 bp, 299 bp
Syne2	TCATGAAGACGACGAGGAGG	CTCAGTCCTGTCACCTTCCA	139 bp, 208 bp
Exoc1	TGAAGATGACTGGCACCTCT	TGTTCCCCATGTCCAGTAGG	156 , 201 bp

*Phactr4 primers detect an extra PCR product, size of 278 bp, from A3SS beside the two predicted PCR products resulted from SE (203 bp and 284 bp).
