# Supplementary material for: An Alternative Splicing Program for Mouse Craniofacial Development
Source: Front Physiol. 2020 Sep 4;11:1099. doi: 10.3389/fphys.2020.01099 (PMC7498679; doi:10.3389/fphys.2020.01099)
Supplement: Supplementary file 13 [file Presentation_1.pdf]

Supplementary Tables and Datasheet

Supplementary Table 1. Gene expression during facial development

Gene-by-sample matrix with the FPKM values called by Cufflinks for all the 60 samples. ecto, ectoderm; epi, epithelium; FNP, frontonasal process; MdP, mandibular prominences; mesen, mesenchyme; MxP, maxillary prominences; NA, a FPKM value was not computed by Cufflinks. Note that this table is not filtered by FPKM.

Supplementary Table 2. Differentially expressed genes during facial development

Differentially expressed genes in this study are defined as genes with cutoffs as: at least one sample with average FPKM >1, maximal linear fold change (MaxFC) > 2, and minimal q value (minQ) < 0.01 from three-way ANOVA analysis. The expression values were log2(FPKM + 1) transformed. The q values (q) from three-way ANOVA analysis for each gene are also shown. a.p.l_q, age.prominence.layer q value; ecto, ectoderm; epi, epithelium; FNP, frontonasal process; Inf, infinite (This occurs when the minimal expression across all samples is zero.); MdP, mandibular prominences; mesen, mesenchyme; MxP, maxillary prominences; NA, a FPKM value was not computed by Cufflinks; prom, prominence.

Supplementary Table 3. Annotation of the DE gene programs

Differentially expressed genes were classified into seven DE gene programs (Late, Pan Ect, Late Mes, Pan Mes, Early Mes, Early Ect, and NE) according to the similarity of their expression patterns (see Figure 1B). Gene annotation of these programs was then performed using Enrichr (Chen et al. 2013; Kuleshov et al. 2016). Additional tabs show the enriched GO Biological Process, Molecular Function, and KEGG 2019 mouse pathways of these gene programs if p-value < 0.001. Note that there were no terms enriched in the Late program when the full list of >2000 genes was employed. After manual curation by removing potential pseudogenes, a list of 697 genes (Curated Late) were used for annotation.

Supplementary Table 4. Summary table of differentially used alternative splicing events called by rMATS by category

Differentially used alternative splicing events in this study are defined as events with FDR < 0.05, |ΔPSI| >= 0.1, and at least one of the average inclusion counts or average skipping counts equal to, or larger than, 5. 45 comparisons were made across age, layer, and prominence (Supplementary Figure 5) for each category of splicing. Events and associated gene numbers are shown for the events with higher PSI in sample 1 or sample 2, while type of comparison is listed and color coded (yellow for across layer comparisons, green for across age comparisons, red for across prominence

comparisons, light yellow for nasal epithelium layer comparisons, and light green for nasal epithelium age comparisons). Different tabs show: SE, skipped exons; A3SS, alternative 3’ splice sites; A5SS, alternative 5’ splice sites; MXE, mutually exclusive exons; RI, retention of intron. Ect, ectoderm; FNP, frontonasal process; MdP, mandibular prominences; Mes, mesenchyme; MxP, maxillary prominences; NE, nasal epithelium; PSI, percentage spliced inclusion.

Supplementary Table 5. AS merged from 45 comparison with max∆PSI cutoff 0.1

Mean PSI from three biological replicates were concatenated from unfiltered rMATS result from all the comparisons (see Supplementary Figure 5). After concatenation, maximal ∆PSI (max∆PSI) was calculated as the difference between the maximal PSI and minimal PSI among the 20 samples for each event. Only the events with max∆PSI > 0.1 were included in this Supplementary table. Different tabs show: SE, skipped exons; A3SS, alternative 3’ splice sites; A5SS, alternative 5’ splice sites; MXE, mutually exclusive exons; RI, retention of intron. Chr, chromosome; ES, exon Start; EE, exon End. Ect, ectoderm; FNP, frontonasal process; MdP, mandibular prominences; Mes, mesenchyme; MxP, maxillary prominences; NA, a PSI was not computed; NE, nasal epithelium; PSI, percentage spliced inclusion

Supplementary Table 6. Annotations of SE events from comparisons without NE samples

The Table contains 4 tabs. The first tab shows the associated genes of differentially used SE events merged from comparisons across age, layer, and prominence. The second tab shows shared or unique associated genes for the overlapped as well as the unique events among these three types of comparisons. The third and fourth tabs, respectively, show the enriched GO Biological Process and Molecular Function terms derived from these gene lists for p-value < 0.001.

Supplementary Table 7. Annotations of shared SE events of layer or age

The Table contains 3 tabs. The first tab shows a list of genes associated with layer or age that fulfil specific criteria. With respect to layer only events that occurred in all three prominences at a specific age were chosen. Subsequently, these individual layer event lists from all three ages were combined together to generate a list of genes that showed consistent AS between ectoderm and mesenchyme in all three prominences at one or more ages (“shared across layer” events). Similarly, for age, events were selected for a particular tissue layer only if they occurred in the ectoderm or mesenchyme of all three prominences between two time points. Subsequently the E10.5_vs_E11.5 and E11.5_vs_E12.5 event lists for ectoderm or mesenchyme were combined together to generate a list of genes that showed consistent AS within the ectoderm or mesenchyme in all three prominences between one or two time points (“shared across age” events). The second tab shows the enriched GO Biological Process, GO Molecular Function and

KEGG 2019 Mouse Pathways terms derived from the “shared across layer” gene lists for p-value < 0.001, and the third tab shows a similar analysis for “shared across age.”

Supplementary Table 8. Annotations of SE layer and age comparisons involving the nasal epithelium

The first tab in the table shows associated genes of SE events merged from four “across layer comparisons” (NE.layer_genes) or two “across age comparisons” (NE.age_genes) involving the nasal epithelium. These gene lists were used for gene annotation with Enrichr (Chen et al. 2013; Kuleshov et al. 2016). The second tab shows the enriched GO Biological Process and Molecular Function of these gene lists for p-value < 0.001.

Supplementary Table 9. Primers and probes used for validation

Supplementary Table 10. Differentially expressed RNA binding proteins during facial development

Differentially expressed RNA binding proteins (RBPs) in this study are defined as RBPs with cutoffs as: at least one sample with average FPKM >1, maximal linear fold change (MaxFC) > 2, and minimal q value (minQ) < 0.01 from three-way ANOVA analysis. The expression values are log2(FPKM + 1) transformed. The q values (q) from three-way ANOVA analysis for each gene are also shown. a.p.l_q, age.prominence.layer q value; ecto, ectoderm; epi, epithelium; FNP, frontonasal process; Inf, infinite (This occurs when the minimal expression across all samples is zero.); MdP, mandibular prominences; mesen, mesenchyme; MxP, maxillary prominences; NA, a FPKM value was not computed by Cufflinks; prom, prominence.

Supplementary Table 11. Differentially expressed splicing regulators during facial development

Differentially expressed splicing regulators in this study are defined as those with cutoffs as: at least one sample with average FPKM >1, maximal linear fold change (MaxFC) > 2, and minimal q value (minQ) < 0.01 from three-way ANOVA analysis. The expression values are log2(FPKM + 1) transformed. The q values (q) from three-way ANOVA analysis for each gene are also shown. a.p.l_q, age.prominence.layer q value; ecto, ectoderm; epi, epithelium; FNP, frontonasal process; Inf, infinite (This occurs when the minimal expression across all samples is zero.); MdP, mandibular prominences; mesen, mesenchyme; MxP, maxillary prominences.

Supplementary Datasheet 1. Filtered rMATS results

This contains a set of different folders relating to: SE, skipped exons; A3SS, alternative 3' splice sites; A5SS, alternative 5' splice sites; MXE, mutually exclusive exons; RI, retention of intron. Each folder contains 45 different comparisons across age, layer, and prominence (see Supplementary Figure 5). Differentially used alternative splicing events in this study are defined as events with FDR < 0.05, $|\Delta PSI| >= 0.1$, and at least one of the average inclusion counts or average skipping counts equal to, or larger than, 5. Chr, chromosome; ES, exon Start; EE, exon End; IC, inclusion counts; SC, skipping counts; IncFormLen, inclusion form length; SkipFormLen, skip form length; IncLevel1, inclusion level of sample 1; IncLevel2, inclusion level of sample 2; DeltaPSI or ΔPSI, difference of percentage spliced inclusion between two samples. Ect, ectoderm; FNP, frontonasal process; MdP, mandibular prominences; Mes, mesenchyme; MxP, maxillary prominences; NE, nasal epithelium; PSI, percentage spliced inclusion.

Supplementary Figures

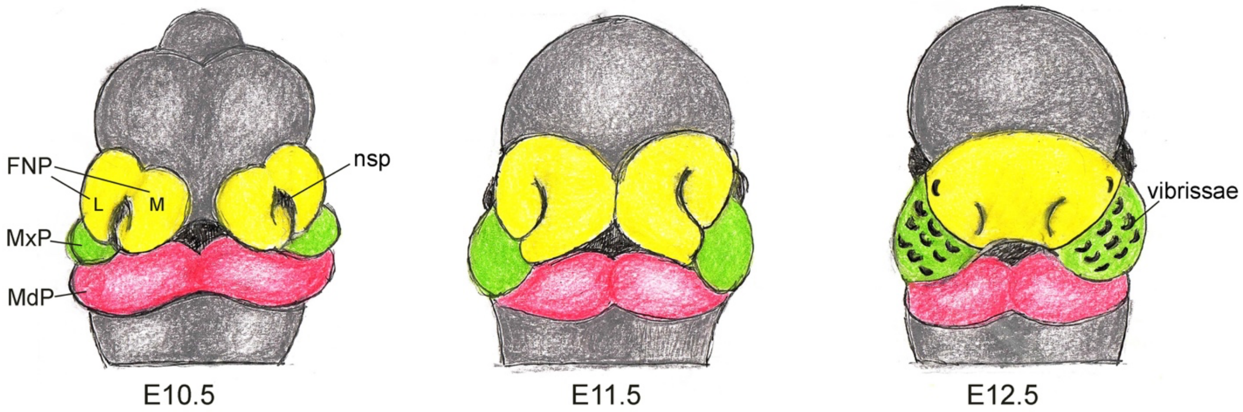

Supplementary Figure 1. Schematic frontal view of mouse face at E10.5, E11.5 and E12.5.

Cartoons (not to scale) show development and morphogenesis of the facial prominences. The regions dissected and analyzed in this study are colored as yellow for FNP, green for MxP, and fuschia for MdP. Over the time course of the analysis, the FNP gives rise to lateral and medial nasal processes, coincident with invagination of the nasal pits. The "FNP" sample always contained both the medial and lateral nasal process tissues combined.

FNP, frontonasal prominence; L, lateral nasal process; M, medial nasal process; MdP, mandibular prominence; MxP, maxillary prominence; nsp, nasal pit.

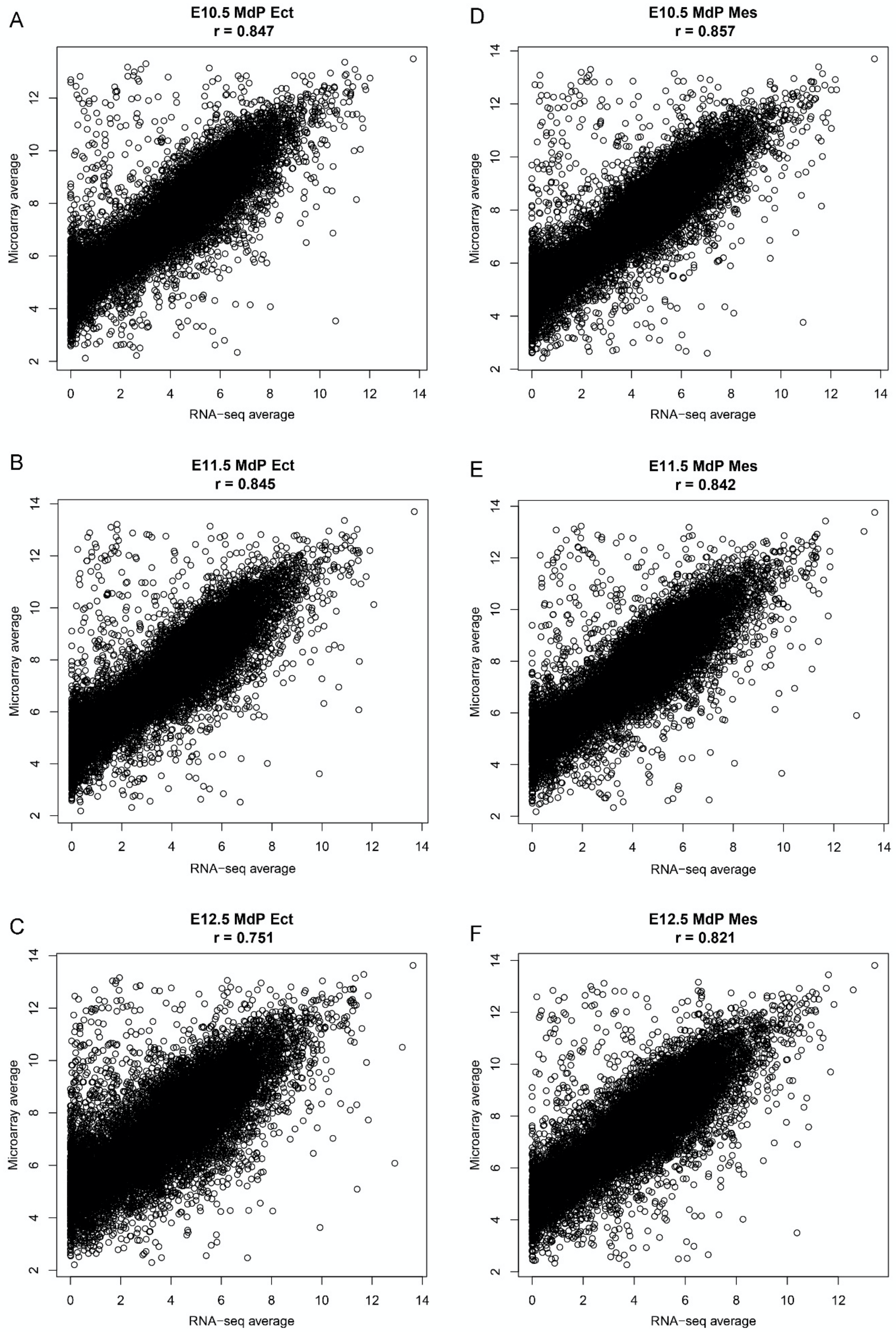
A
E10.5 MdP Ect
r = 0.847
D
E10.5 MdP Mes
r = 0.857
B
E11.5 MdP Ect
r = 0.845
E
E11.5 MdP Mes
r = 0.842
C
E12.5 MdP Ect
r = 0.751
F
E12.5 MdP Mes
r = 0.821
Microarray average
RNA−seq average

Supplementary Figure 2. High correlation of RNA-seq data with previous microarray data.

Scatter plots show high agreement across the RNA-seq data and previously published microarray data (GEO: GSE62214) from equivalent samples (Hooper et al. 2017) for all the mandibular prominence tissues (A-F). For each plot, the tissue name is shown on top with Pearson coefficient (r). The x-axis indicates the average expression of genes in the RNA-seq data from three biological replicates at log2 scale, and the y-axis indicates the average signal of genes in the microarray data from three biological replicates at log2 scale. Only genes detected in both datasets were plotted. For the microarray data, if there was more than one probe for a gene, the average signal from all probes was used. MdP, mandibular prominence; Ect, ectoderm; Mes, mesenchyme.

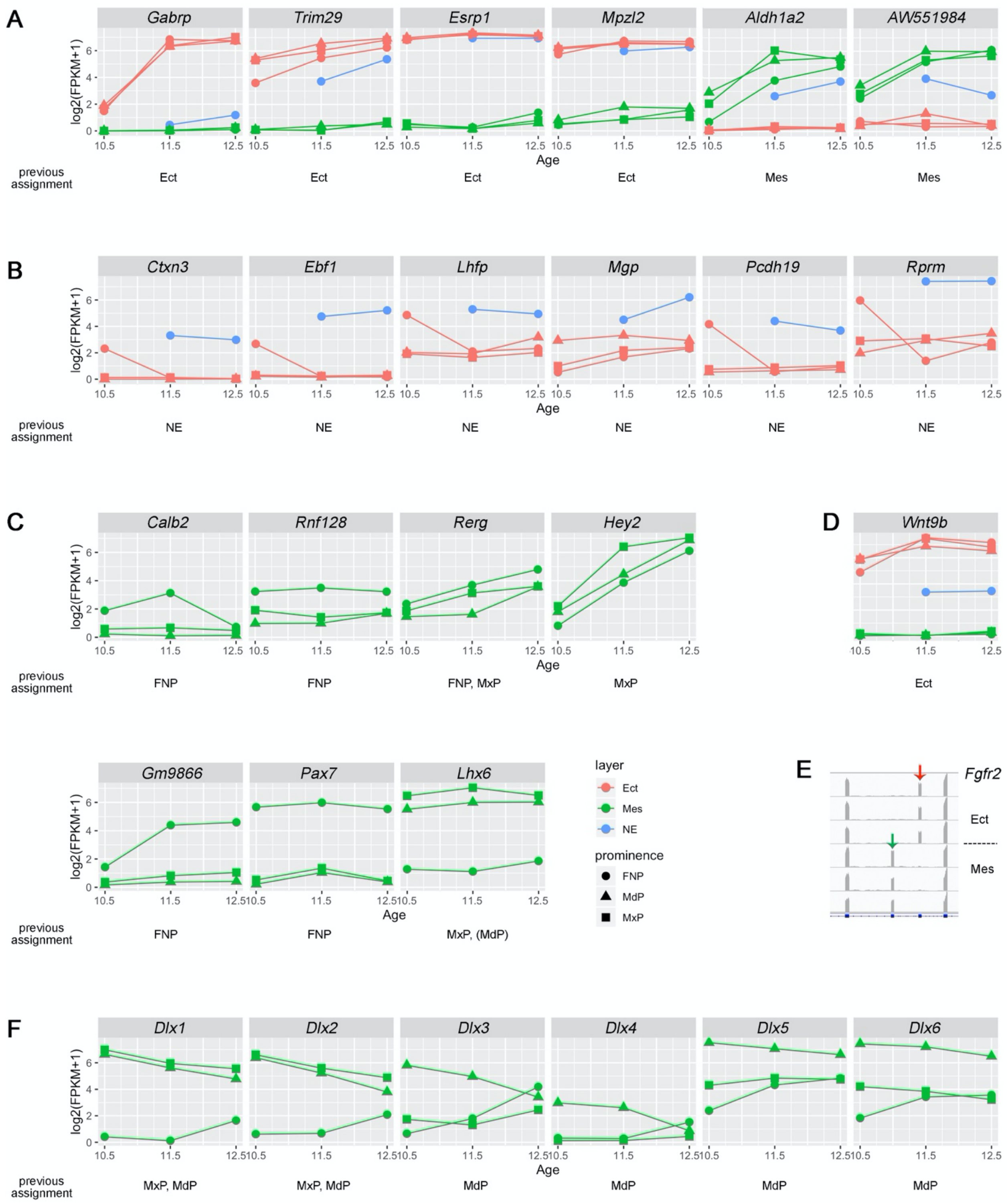

Supplementary Figure 3. Fidelity of sample preparation and consistency of the RNA-seq data with previous microarray and single cell RNA-seq data.

(A) Expression profiles of six genes from the current RNA-seq dataset that were previously validated and assigned to specific tissue layers by in situ hybridization

following microarray analysis (See Figure 2A in (Hooper et al. 2017)). In general, there is excellent agreement between the expression profiles and assignments from the two studies, but by separating the E11.5 and E12.5 FNP into three samples - the mesenchyme, surface ectoderm and nasal epithelium, a more accurate picture of gene expression in the FNP mesenchyme and NE can be determined (illustrated by the consistent expression patterns of *Esrp1* and *Mpzl2* across the various prominences compared to higher expression in the FNP mesenchyme in the previous microarray data, as well as the lower expression of *Gabrp* and *Trim29* in the NE in contrast to the surface ectoderm).

(B) Expression profiles of six olfactory epithelial marker genes from the current RNA-seq dataset that were assigned to the same tissue layer in a previous single cell RNA-seq study (Li et al. 2019). Note that only the Ect and NE samples are shown here for simplicity.

(C) Expression profiles of seven genes with differential expression across the facial prominences from the current RNA-seq dataset that were assigned to the same prominences in a previous single cell RNA-seq study of the upper face followed by *in situ* hybridization (Li et al. 2019). Note that only the Mes samples are shown here for simplicity. Also note that although we show expression in all three prominences for the current RNA-seq dataset, only the MxP and FNP were analyzed in the single cell RNA-seq study. This mainly affects the "previous assignment" of *Lhx6* and we have added MdP in parentheses to highlight this discrepancy.

(D) Expression profile of *Wnt9b* from the current RNA-seq dataset, a known surface ectoderm marker that was also located in this layer using single cell RNA-seq (Li et al. 2019).

(E) Tissue-specific splicing differences between ectoderm and mesenchyme detected in the current RNA-seq datasets. Triplicate RNA-seq samples derived from either the ectoderm or mesenchyme of the E11.5 MxP shown as screenshots taken from the IGV browser. *Fgfr2* shows mutually exclusive exon usage (arrows) between the ectoderm and mesenchyme, corresponding the to the *Fgfr2* IIIb and IIIc isoforms, respectively. Note that the tracks are auto-scaled, therefore the heights of the tracks are not scaled to the expression of *Fgfr2*.

(F) Expression profiles of Dlx genes across the facial prominences from the current RNA-seq dataset that were assigned to the same prominences in a previous microarray dataset of whole facial prominences (Feng et al. 2009). These profiles are consistent with the established expression patterns of Dlx genes by whole mount *in situ* hybridization (Minoux and Rijli 2010). Note that only the Mes samples are shown here for simplicity.

The expression of the established marker genes and splicing events for tissue layers (e.g. *Esrp1*, *Wnt9b*, and *Fgfr2*) and prominences (e.g. *Pax7* for FNP, Dlx genes for *MdP* and *MxP*) demonstrate the fidelity of the RNA-seq samples.

In each panel of (A-D, F), the x axis shows the embryonic stages, and y axis shows the average expression (log2 scale) of the sample from three biological replicates. The previous assignment from the microarray datasets or single cell RNA-seq dataset is shown at the bottom of each panel. Layers are shown with colors (red for Ect, green for Mes, and blue for NE), while prominences are distinguished by shapes (circle for FNP, triangle for MdP, and rectangle for MxP). Ect, ectoderm; FNP, frontonasal process; MdP, mandibular prominence; Mes, mesenchyme; MxP, maxillary prominence; NE, nasal epithelium.

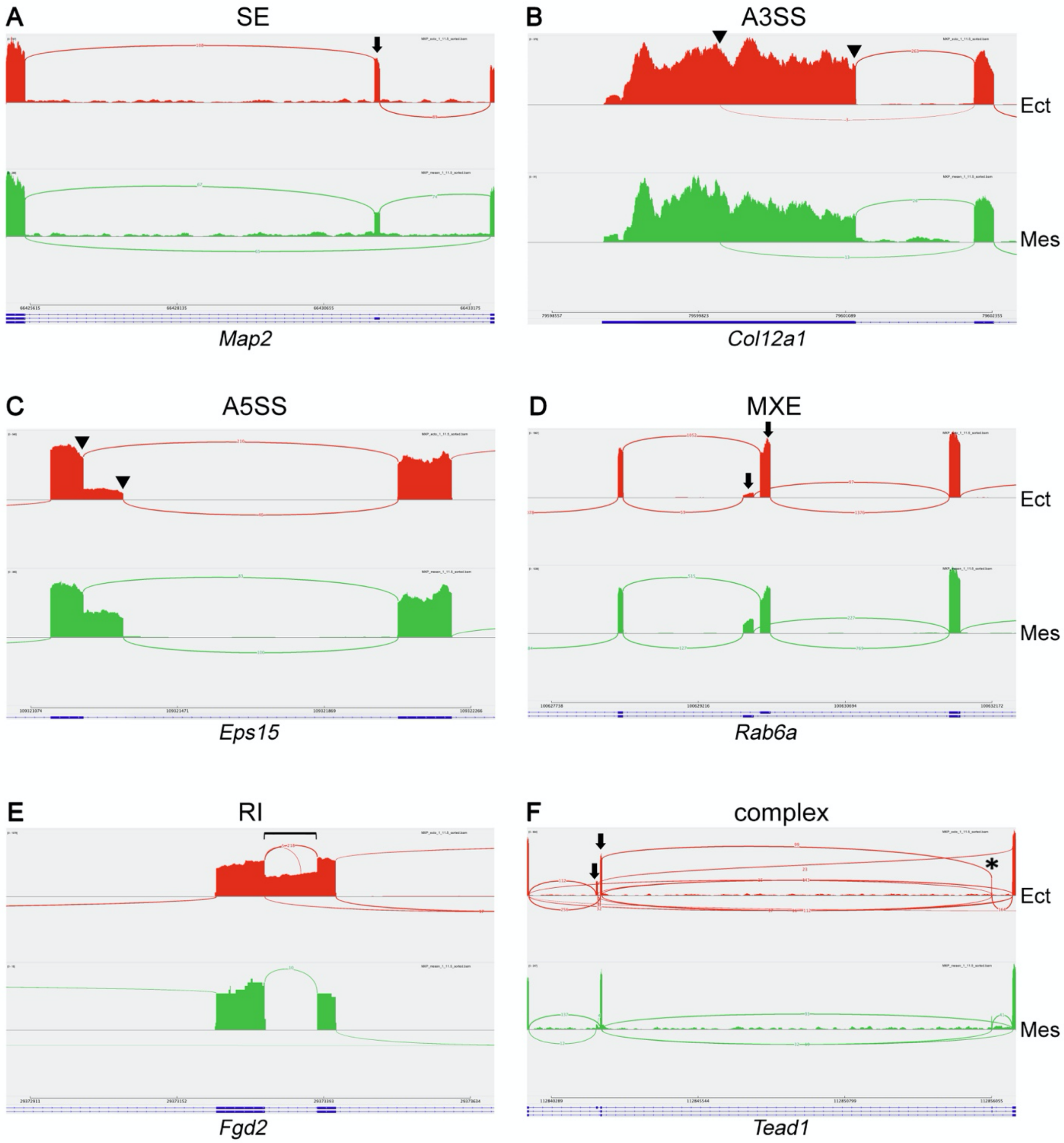

Supplementary Figure 4. Examples of categories of alternative splicing.

Sashimi plots show examples of skipped exon (A), alternative 3' splice site (B), alternative 5' splice site (C), mutually exclusive exon (D), retained intron (E), and complex alternative splicing (F) that was called as MXE by rMATS.

In each sashimi plot, the top track is the RNA-seq data of E11.5 MxP ectoderm sample 1 (red), while the bottom track is the RNA-seq data of E11.5 MxP mesenchyme sample 1 (green). Isoform models are shown under these tracks with blue rectangles as exons, lines as introns, and arrowheads indicating the direction of transcripts. Note that the

direction of *Col12a1* (B) is from right to left. The type of alternative splicing is shown on the top and the gene name on the bottom for each panel. The vertical piles are RNA-seq reads of exons, while the arcs connecting exons show how the exons are spliced (splicing junctions). Arrows point to the differentially used exons, while triangles indicate the alternative splicing sites. Bracket in E shows the retained intron region. Asterisk in F indicates the additional differential used exon in the complex example.

A3SS, alternative 3’ splice sites; A5SS, alternative 5’ splice sites; Ect, ectoderm; Mes, mesenchyme; MXE, mutually exclusive exons; MxP, maxillary prominence; SE, skipped exon; RI, retained introns.

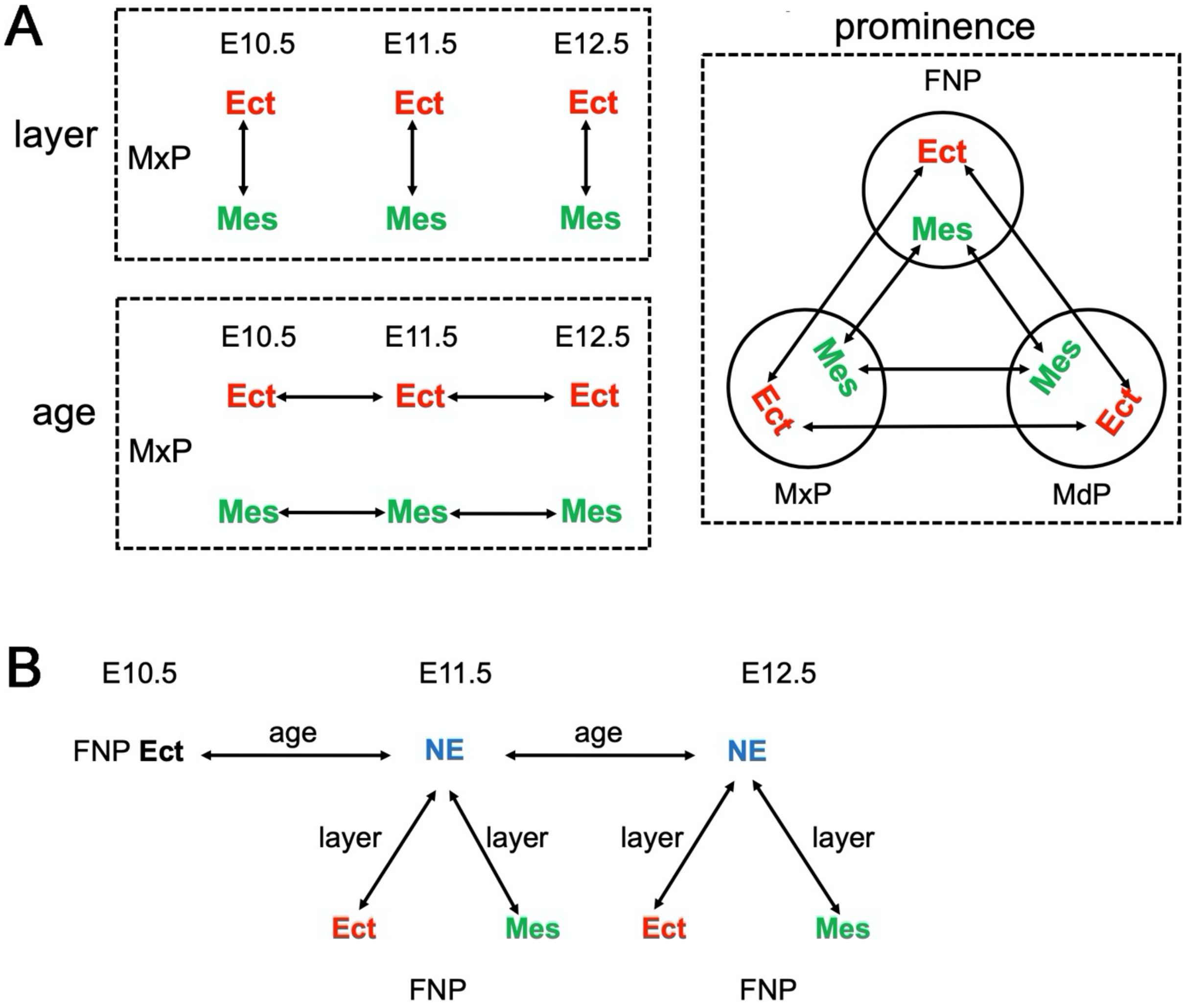

Supplementary Figure 5. rMATS comparisons.

Individual tissue comparisons from the rMATS analysis are indicated by the double headed arrows. (A) The three dotted rectangles show three types of comparison: across layer, age, or prominence – but excluding the nasal epithelium samples. Examples are show for across layer comparisons for the MxP at three stages, across age comparisons for the MxP tissues layers Ect and Mes, and an across prominence comparison at one age. In total, there are 9 comparisons across layer, 12 comparisons across age, and 18 comparisons across prominence. (B) Across age and layer comparisons involving nasal epithelium samples. The type of comparison is marked near the double head arrows. There are four across layer comparisons and two across age comparisons in total.

Ect, ectoderm; FNP, frontonasal process; MdP, mandibular prominences; Mes, mesenchyme; MxP, maxillary prominences; NE, nasal epithelium.

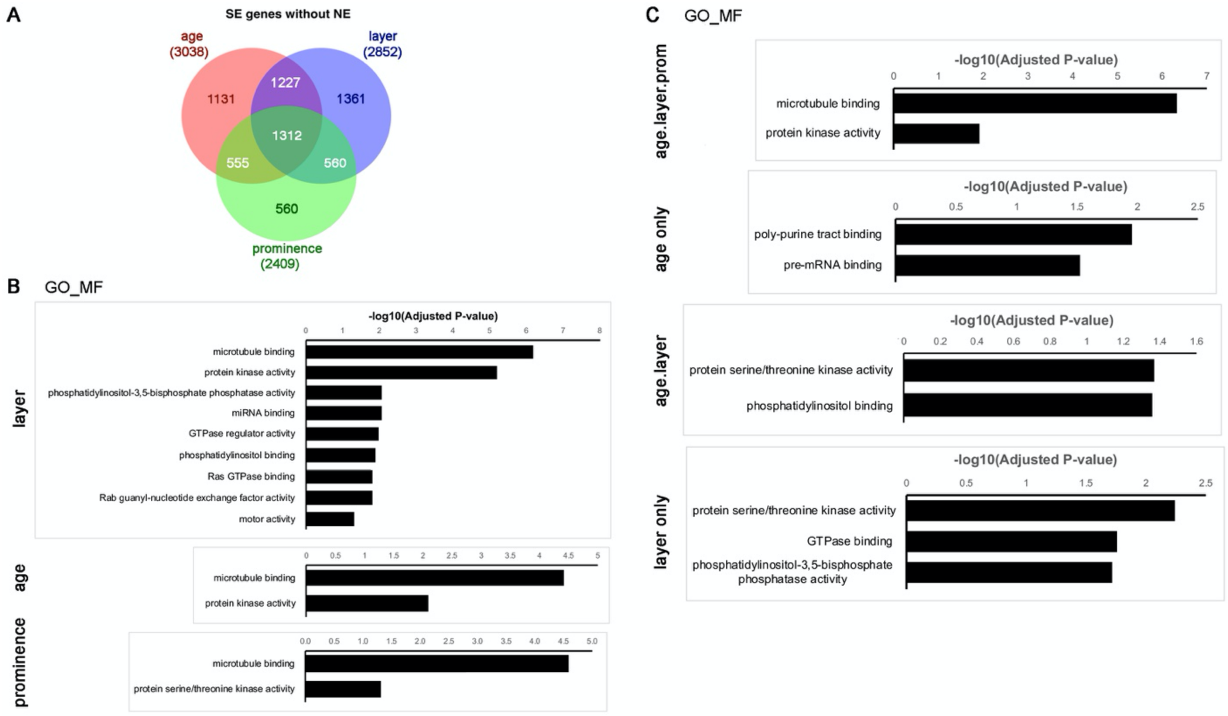

Supplementary Figure 6. Gene annotation of differentially used SE events during facial development.

(A) Venn diagram showing the gene numbers corresponding to the differentially used SE events (as shown in Fig 3A) called from across age, layer, and prominence comparisons, but omitting the nasal epithelium (NE) samples. The numbers of shared or unique genes are marked in the corresponding area.

(B) GO Molecular Function terms (GO_MF) enriched for the associated SE genes across layer, age, and prominence comparisons with adjusted p-value < 0.05. Each bar graph shows the -log10(adjusted p-value) for enriched gene ontologies as listed on the left. Note that redundant terms were removed manually before plotting.

(C) GO Molecular Functions (GO_MF) enriched for the associated genes of SE events shared by age, layer, and prominence (age.layer.prom), used only by age, shared by age and layer (age.layer), and used only by layer with adjusted p-value < 0.05. Each bar graph shows the -log10(adjusted p-value) for enriched gene ontologies as listed on the left. Note that redundant terms were removed manually before plotting and there were no terms enriched in the associated genes of SE events used only by prominence, shared by age and prominence, or shared by layer and prominence.

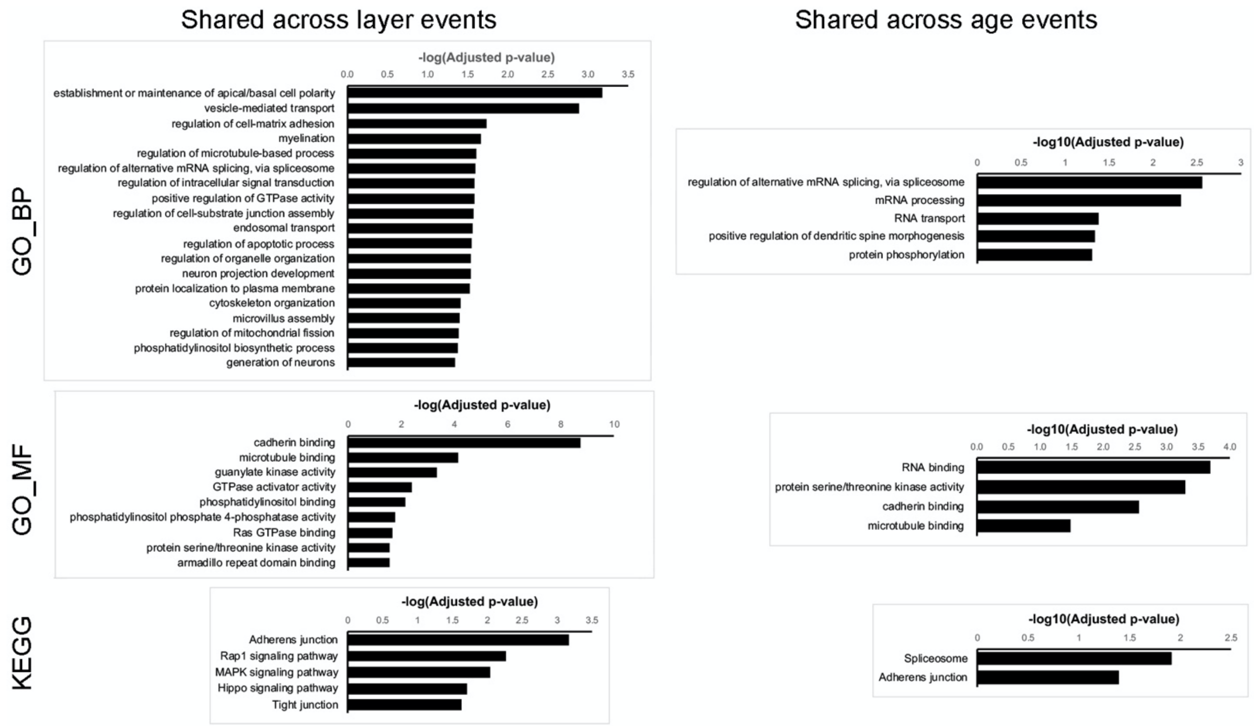

Supplementary Figure 7. Gene annotation of shared SE events from layer and age comparisons.

Genes associated with differentially used “shared across layer” (left) or “shared across age” (right) SE events, as shown in Supplementary Table 7, were subject to Enrichr analysis (Chen et al. 2013; Kuleshov et al. 2016) (https://amp.pharm.mssm.edu/Enrichr/). GO term biological process (GO_BP, top panels), molecular function (GO_MF, middle panels), and KEGG pathway (KEGG, bottom panels) are shown for those with adjusted p-value < 0.05. Each bar graph shows the -log10(adjusted p-value) for enriched gene ontologies as listed on the left. Note that redundant terms were removed manually before plotting and “shared across prominence” was not included in this analysis because there were only a few “shared across prominence” events.

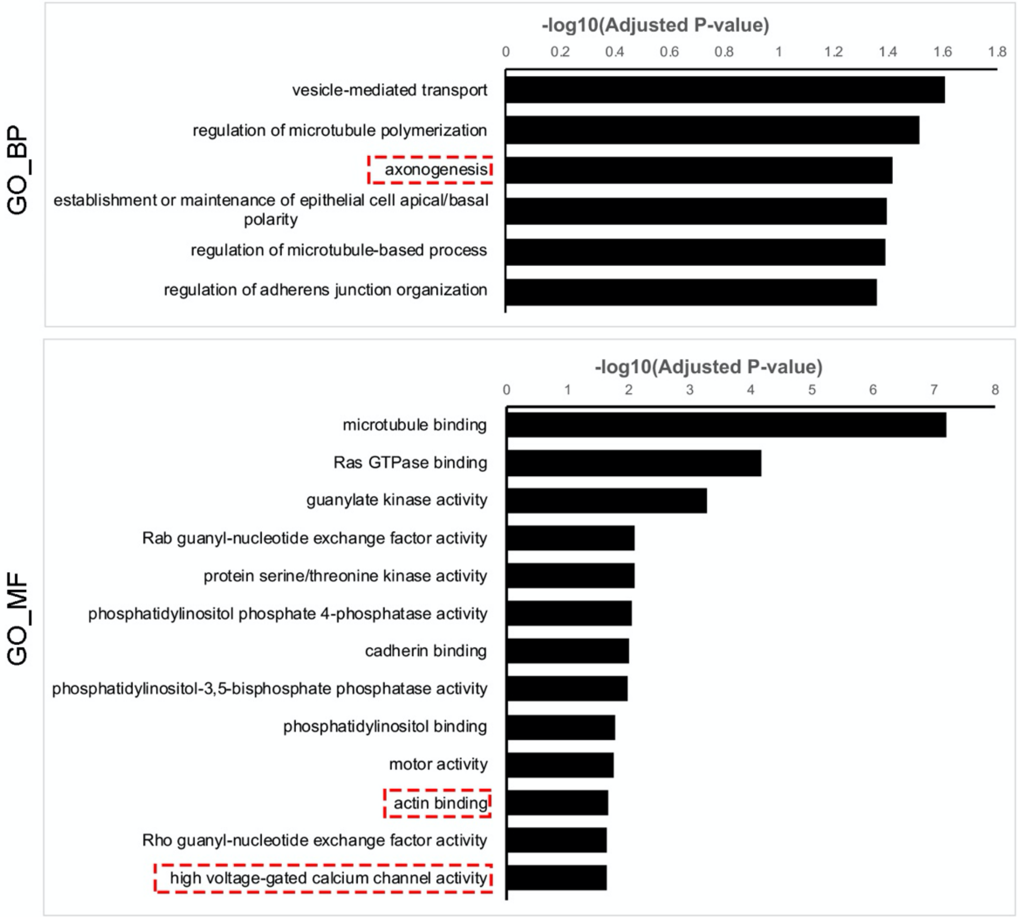

Supplementary Figure 8. Gene annotation of SE genes from nasal epithelium across layer comparisons.

Genes associated with differentially used SE events derived from layer comparisons involving nasal epithelium (NE) tissues, as shown in Supplementary Table 8, were subject to Enrichr analysis (Chen et al. 2013; Kuleshov et al. 2016) for gene annotation (https://amp.pharm.mssm.edu/Enrichr/). Significant (adjusted p-value < 0.05) GO biological process (GO_BP) and molecular function (GO_MF) terms are shown in the top and bottom panels, respectively. Each bar graph shows the -log10(adjusted p-value) for enriched gene ontologies as listed on the left. The red dotted rectangles highlight the ontologies specifically enriched related to neurons and/or neurogenesis. Note that redundant terms were removed manually before plotting and there was no enrichment for KEGG pathways.

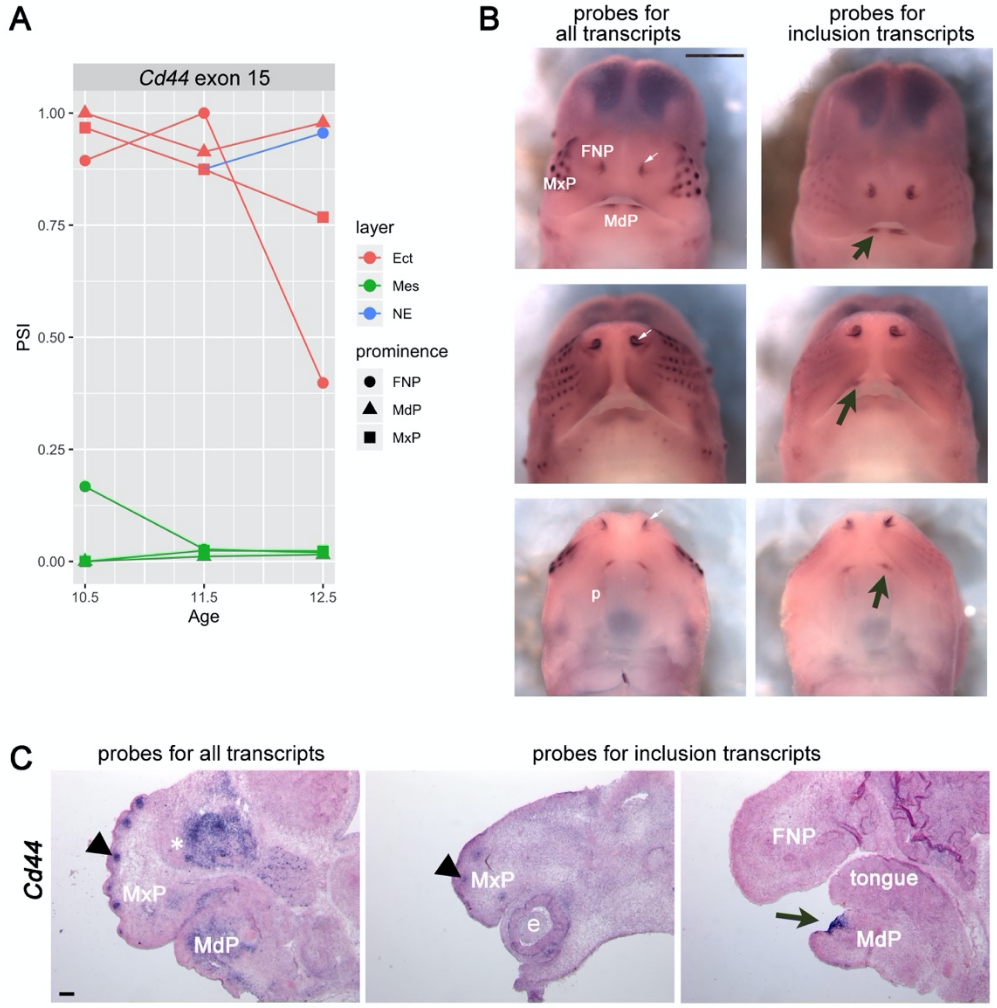

Supplementary Figure 9. *In situ* hybridization of *Cd44*.

(A) Usage of *Cd44* exon 15 (see Figure 4A) is shown during facial development in a Percentage of splicing inclusion (PSI) plot with the x axis representing age and the y axis PSI. Layers are shown by colors (red for Ect, green for Mes, and blue for NE), while prominences are distinguished by shapes (circle for FNP, triangle for MdP, and rectangle for MxP).

(B) Whole mount *in situ* hybridization of E12.5 embryos for probes detecting all *Cd44* transcripts (left panels) or probes specific for ectodermal transcripts including exon 6 to exon 15 (right panels) (see Figure 4B). Frontal views (top panels), frontal views with head slightly tilted towards to the back (middle panels), and palatal views with removed mandible (bottom panels) are shown. The small white arrows point to the nasal cavity, while the large black arrows point to the expression of *Cd44* in tooth buds detected by probes specific for ectodermal transcripts. Scale bar, 1 mm.

(C) *In situ* hybridization of *Cd44* on E12.5 facial sagittal sections. Probes detecting all *Cd44* transcripts (left panel) and probes detecting ectodermal transcripts

including exon 6 to exon 15 (middle and right panels) were used (see Figure 4A). Blue color is the signal for *in situ* hybridization. The sections were counterstained with nuclear fast red. Arrowheads indicate vibrissae, while the asterisk shows an example of mesenchymal expression of *Cd44* detected by probes for all transcripts. The large black arrow points to the expression of *Cd44* in tooth buds detected by probes specific for ectodermal transcripts. Scale bar, 200 uM.

e, eye; Ect, ectoderm; FNP, frontonasal process; MdP, mandibular prominence; Mes, mesenchyme; MxP, maxillary prominence; NE, nasal epithelium; p, palatal shelf.

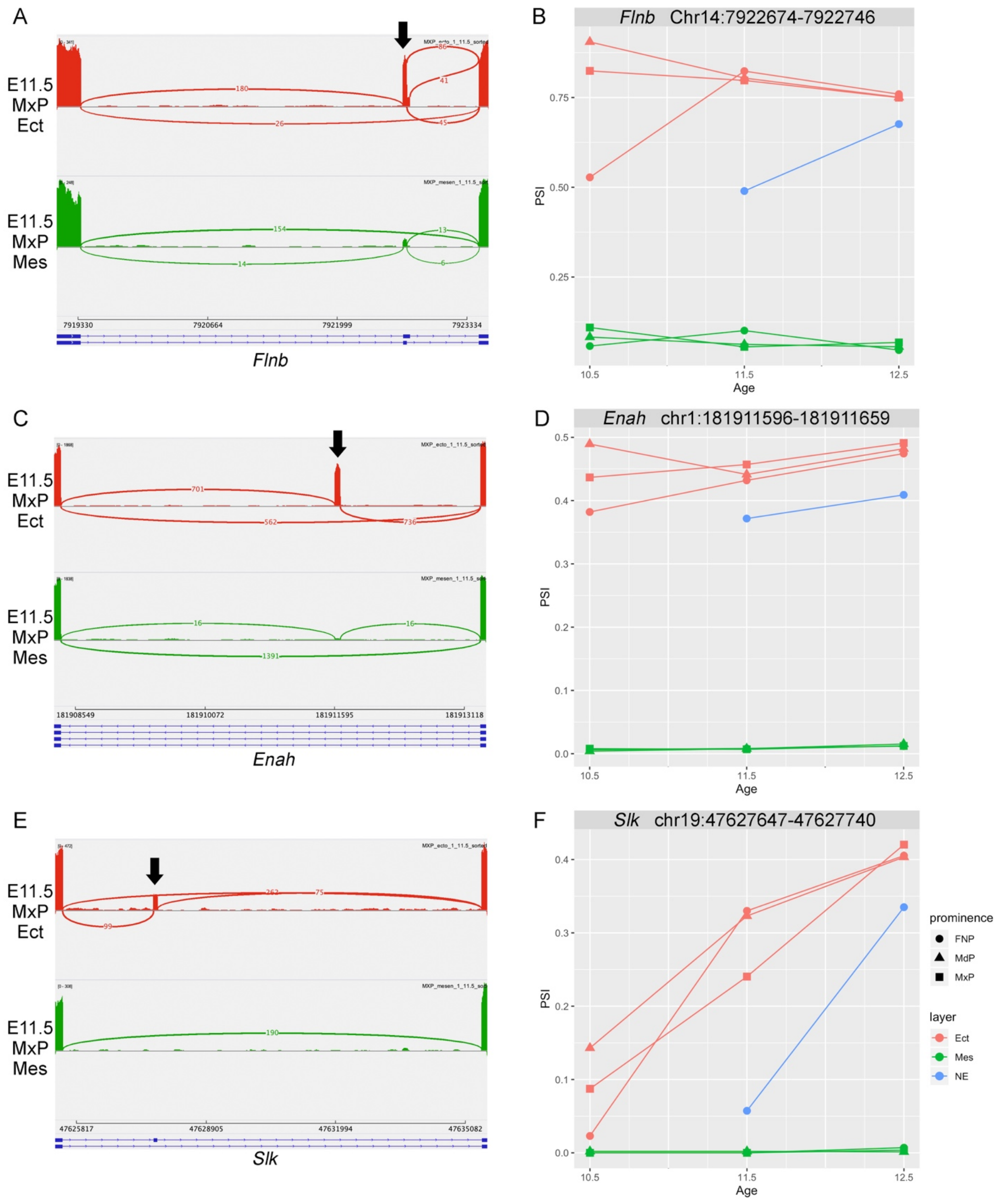

Supplementary Figure 10. SE events used for Basescope.

Skipped exon differential usage data used for the design of Basescope probes for analysis of the tissue specific expression of *Flnb* (A, B), *Enah* (C, D), and *Slk* (E, F) during facial development. (A), (C), and (E) are sashimi plots in which the top track is

the RNA-seq data of E11.5 MxP ectoderm sample 1 (red), and the bottom track is the RNA-seq data of E11.5 MxP mesenchyme sample 1 (green). Isoform models are shown under these tracks with blue rectangles as exons, lines as introns, and arrowheads indicating the direction of transcription. The gene names are on the bottom. The vertical piles are RNA-seq reads of exons, while the arcs connecting exons show the splicing junctions. Arrows point to the differentially skipped exons. (B), (D), and (F) show the corresponding PSI plot, in which the x axis shows the age, and y axis shows the percentage of splicing inclusion (PSI) for the skipped exon. The gene name is shown on top, alongside the chromosomal location of the skipped exon. Layers are shown by colors (red for Ect, green for Mes, and blue for NE), while prominences are distinguished by shapes (circle for FNP, triangle for MdP, and rectangle for MxP).

Ect, ectoderm; FNP, frontonasal process; MdP, mandibular prominences; Mes, mesenchyme; MxP, maxillary prominences; NE, nasal epithelium.

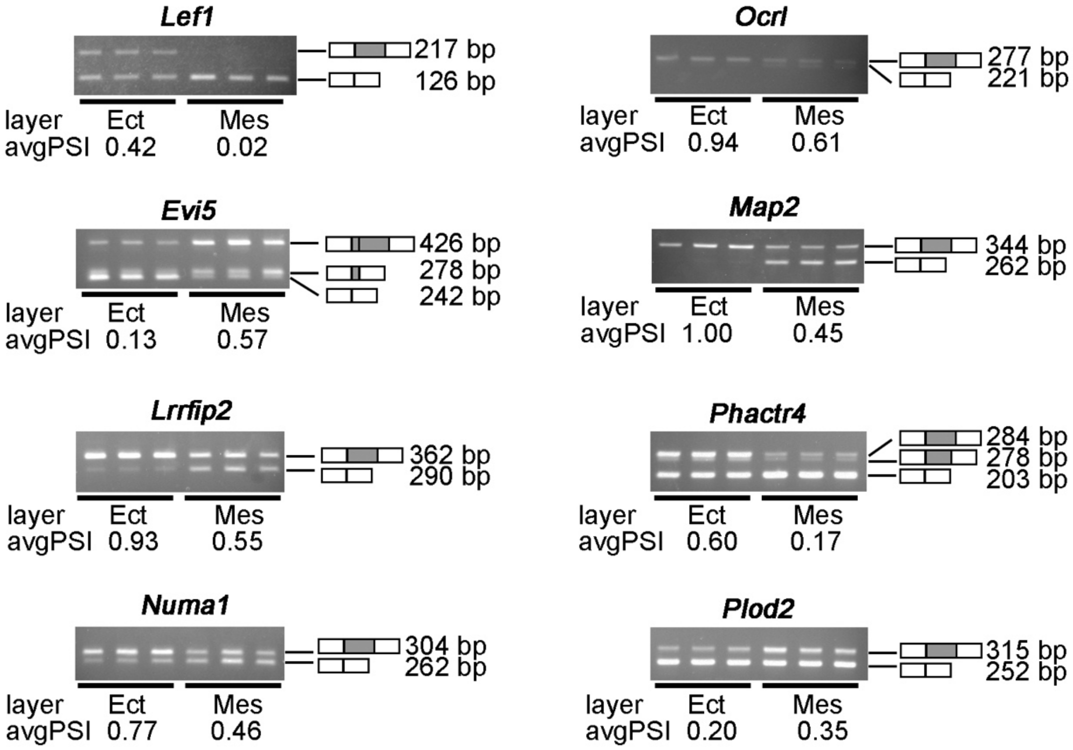

Supplementary Figure 11. RT-PCR validation of differentially used SE events across layer.

Differentially utilized skipped exon occurrences for eight genes, called by rMATS from comparison of E11.5 maxillary prominence ectoderm (Ect) and mesenchyme (Mes), were validated by reverse transcription PCR (RT-PCR). The primers were designed to span the skipped exon to detect the isoforms of both skipping the exon and including the exon. In each panel, the gel image of the PCR products from three biological replicates of Ect and Mes tissues is shown in the center, with gene name on the top, schematic of inclusion and skipping with the sizes of the bands on the right, and annotation of the samples and average PSI (avgPSI) called by rMATS on the bottom.

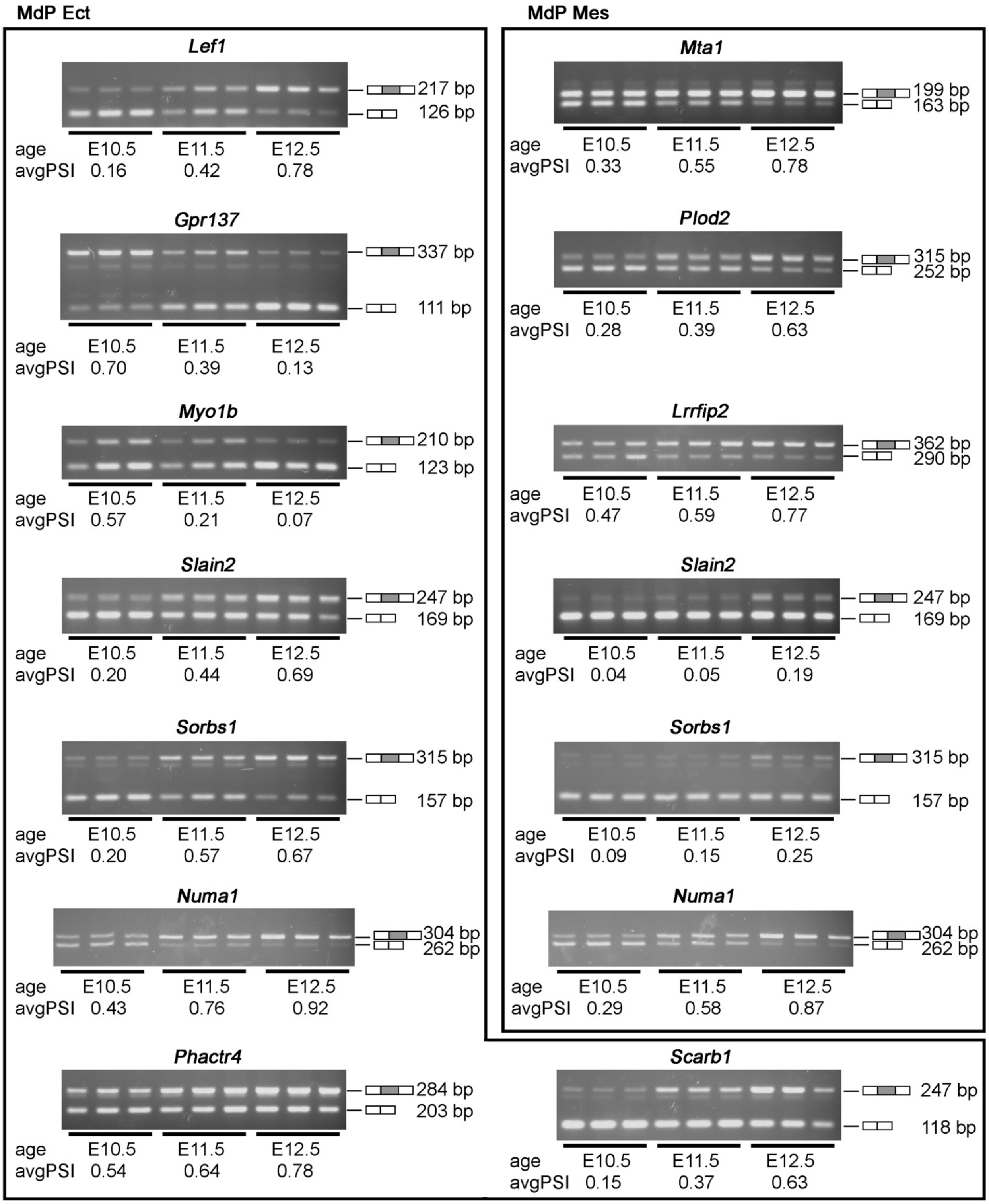

Supplementary Figure 12. RT-PCR validation of differentially used SE events across age.

Differentially utilized skipped exon occurrences called by rMATS from comparison of mandibular prominence ectoderm (MdP Ect) and mandibular mesenchyme (MdP Mes) across three ages (E10.5, E11.5, and E12.5) were validated by RT-PCR. The primers were designed to span the skipped exon to detect the isoforms of both skipping the exon and including the exon. In each panel, the gel image of PCR products from three biological replicates of E10.5, E11.5, and E12.5 is shown in the center, with gene name on the top, schematic of inclusion and skipping with the sizes of the bands on the right, and annotation of the samples and average PSI (avgPSI) called by rMATS on the bottom. Mandibular prominence ectodermal tissues (MdP Ect) were used for all the left panels and the bottom right panel. Mandibular prominence mesenchymal tissues (MdP Mes) were used for the right panels with the exception of the bottom one.

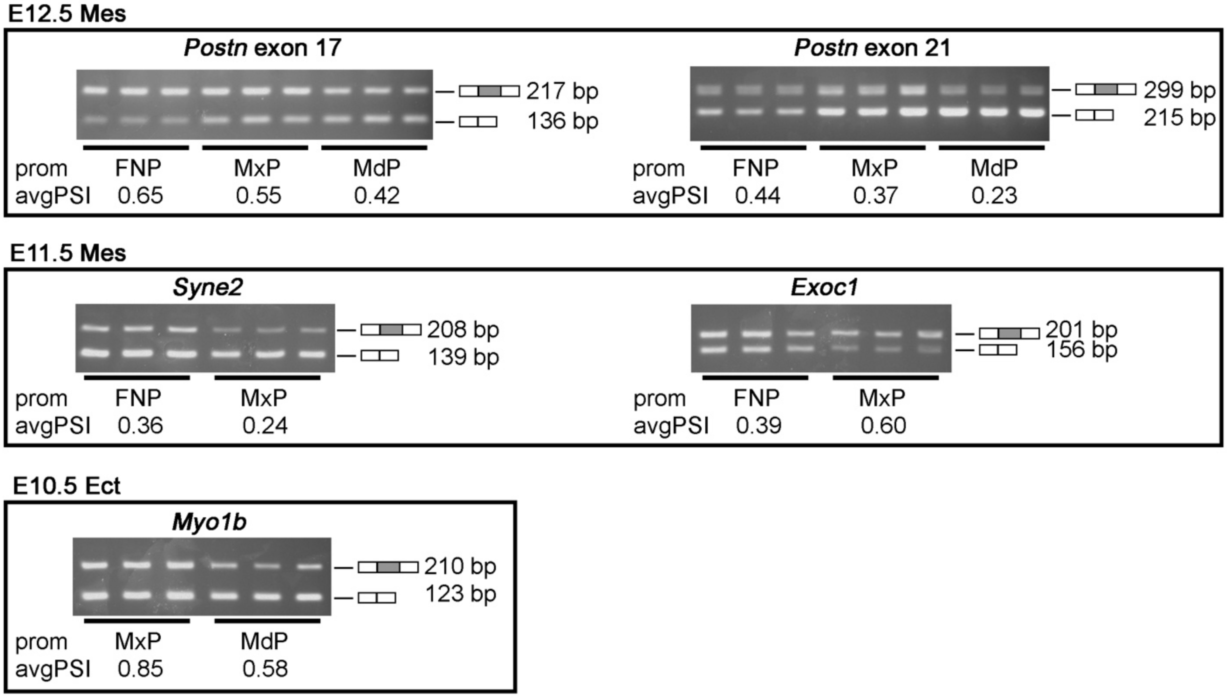

Supplementary Figure 13. RT-PCR validation of differentially used SE events across prominences.

Differentially utilized skipped exon occurrences called by rMATS from comparison across prominences were validated by RT-PCR. The primers were designed to span the skipped exon to detect the isoforms of both skipping the exon and including the exon. In each panel, the gel image of PCR products from three biological replicates of each sample is shown in the center, with gene name, or gene name with exon number, on the top, schematic of inclusion and skipping with the sizes of the bands on the right, and annotation of the samples and average PSI (avgPSI) called by rMATS on the bottom. E12.5 mesenchymal tissues from all three facial prominences (FNP, MxP, and MdP) were used for the top row panels. E11.5 mesenchymal tissues from FNP and MxP were used for the middle row panels. E10.5 ectodermal tissues from MxP and MdP were used for the bottom panel.

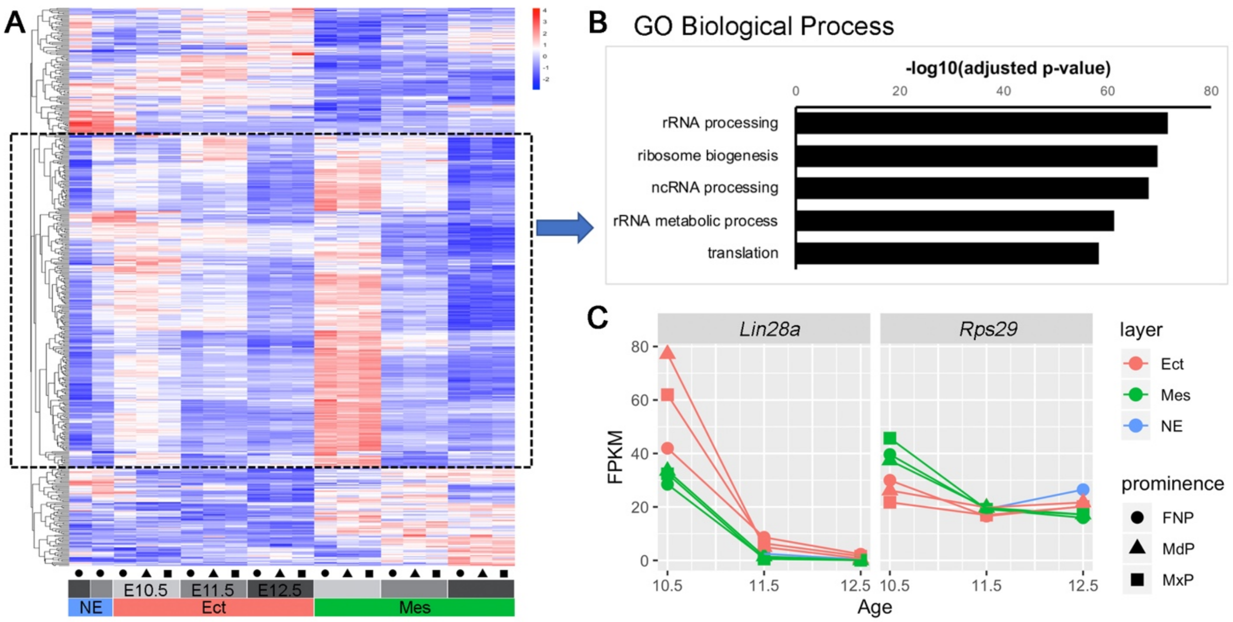

Supplementary Figure 14. Differentially expressed RNA binding proteins during facial development.

(A) Heatmap of differentially expressed RNA binding proteins (RBP). Each row is the scaled expression of an RBP, and each column is the average expression from three biological replicates. Differentially expressed RBPs were defined as at least one sample with average FPMK >1, maximal fold change (MaxFC) > 2, and minimal q value < 0.01 from three-way ANOVA analysis. The columns are annotated at the bottom with colors for tissue layers (red for Ect, green for Mes, and blue for NE), gray intensity for ages (light for E10.5, intermediate for E11.5, and dark for E12.5), and shapes for prominences (circle for FNP, triangle for MdP, and rectangle for MxP). The dotted rectangle indicates a large group of RBPs with decreased expression over age, particularly in the Mes tissues, which have high expression at E10.5 but reduced expression thereafter. (B) Top 5 GO Biological Process terms of the group of RBPs within the dotted rectangle in (A) annotated with Enrichr software (Chen et al. 2013; Kuleshov et al. 2016) (https://amp.pharm.mssm.edu/Enrichr/). The bar graph shows the -log10(adjusted p-value) for the top 5 enriched gene ontologies as listed on the left. (C) Expression of *Lin28a* and *Rps29* during facial development. *Lin28a* and *Rps29* are two examples of the RBPs within the dotted rectangle in A. In each panel, the x axis shows the embryonic stages, and the y axis shows the average FPKM of the sample from three biological replicates. Layers are shown with colors (red for Ect, green for Mes, and blue for NE), while prominences are distinguished by shapes (circle for FNP, triangle for MdP, and rectangle for MxP). Ect, ectoderm; FNP, frontonasal process; MdP, mandibular prominences; Mes, mesenchyme; MxP, maxillary prominences; NE, nasal epithelium.

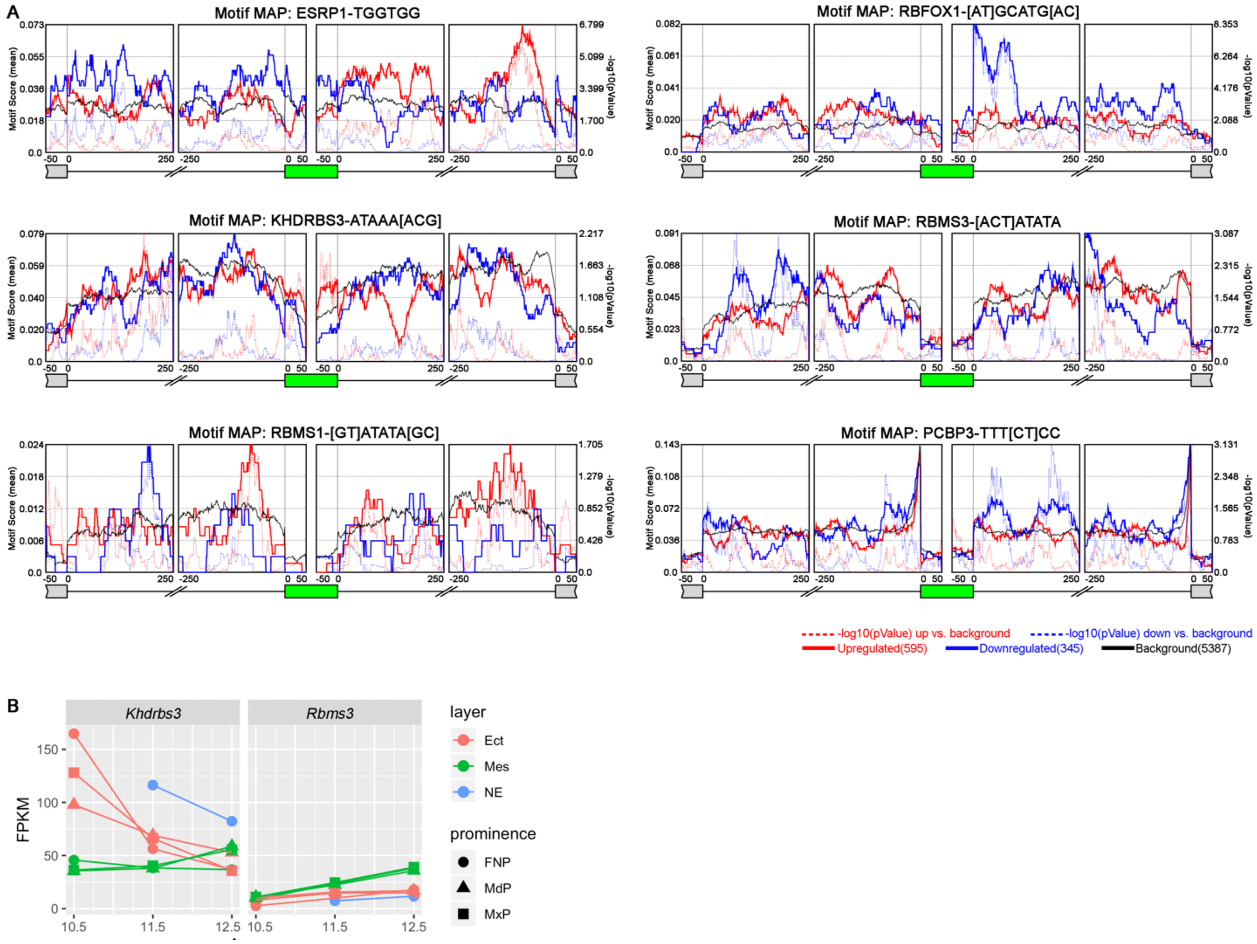

Supplementary Figure 15. Motif enrichment predicted by rMAPS2 for the SE events of the E11.5 MxP Ect vs Mes comparison.

(A) Motif maps for *Esrp1*, *Khdrbs3*, *Rbms1*, Rbfox1, *Rbms3*, and *Pcbp3*, with the motif sequence on the top, graph of predicted motif scores and -log10(pValue) in the middle, and diagram of the SE events on the bottom of each motif map. rMAPS2 (Hwang et al. 2020) (http://rmaps.cecsresearch.org/) was used with the default setting for the SE events from the E11.5 MxP Ect vs Mes comparison. Upregulated refers to SE events with higher PSI in Ect (or lower PSI in Mes), while downregulated indicates SE events with lower PSI in Ect (or higher PSI in Mes). The diagram of the gene shows the skipped exon in green, with the upstream and downstream exons in gray. The motif data is split into four blocks covering 50 nts of an exon and the adjacent 250 nts of the associated intron. In each motif map, the y axis in the left side shows the motif score, while the y axis in the right side shows the -log10(pValue). The red, blue, and black lines indicate the motif scores for upregulated (595), downregulated (345), and background (5387) events, respectively. The red and blue dashed lines indicate the corresponding -log10(pValue) of upregulated events vs background events, and downregulated events vs background events. (B) Expression of *Khdrbs3* and *Rbms3* during facial development. In each panel, the x axis shows the embryonic stages, and y

axis shows the average FPKM of the sample from three biological replicates. Layers are shown with colors (red for Ect, green for Mes, and blue for NE), while prominences are distinguished by shapes (circle for FNP, triangle for MdP, and rectangle for MxP). Ect, ectoderm; FNP, frontonasal process; MdP, mandibular prominences; Mes, mesenchyme; MxP, maxillary prominences; NE, nasal epithelium.

References

Chen EY, Tan CM, Kou Y, Duan Q, Wang Z, Meirelles GV, Clark NR, Ma'ayan A. 2013. Enrichr: Interactive and collaborative HTML5 gene list enrichment analysis tool. BMC Bioinformatics. 14:128.

Feng W, Leach SM, Tipney H, Phang T, Geraci M, Spritz RA, Hunter LE, Williams T. 2009. Spatial and temporal analysis of gene expression during growth and fusion of the mouse facial prominences. PLoS One. 4(12):e8066.

Hooper JE, Feng W, Li H, Leach SM, Phang T, Siska C, Jones KL, Spritz RA, Hunter LE, Williams T. 2017. Systems biology of facial development: Contributions of ectoderm and mesenchyme. Dev Biol. 426(1):97-114.

Hwang JY, Jung S, Kook TL, Rouchka EC, Bok J, Park JW. 2020. rMAPS2: An update of the RNA map analysis and plotting server for alternative splicing regulation. Nucleic Acids Res. 48, W300-W306.

Kuleshov MV, Jones MR, Rouillard AD, Fernandez NF, Duan Q, Wang Z, Koplev S, Jenkins SL, Jagodnik KM, Lachmann A et al. 2016. Enrichr: A comprehensive gene set enrichment analysis web server 2016 update. Nucleic Acids Res. 44, W90-W97.

Li H, Jones KL, Hooper JE, Williams T. 2019. The molecular anatomy of mammalian upper lip and primary palate fusion at single cell resolution. Development. 146: dev174888.

Minoux M, Rijli FM. 2010. Molecular mechanisms of cranial neural crest cell migration and patterning in craniofacial development. Development. 137(16):2605-2621.
